# Researchers’ perceptions of research misbehaviours: a mixed methods study among academic researchers in Amsterdam

**DOI:** 10.1186/s41073-019-0081-7

**Published:** 2019-12-02

**Authors:** Tamarinde L. Haven, Joeri K. Tijdink, H. Roeline Pasman, Guy Widdershoven, Gerben ter Riet, Lex M. Bouter

**Affiliations:** 1 Department of Philosophy, Vrije Universiteit Amsterdam, De Boelelaan 1105, 1081 HV Amsterdam, The Netherlands; 2 Department of Medical Humanities, Amsterdam UMC, Vrije Universiteit Amsterdam, VUmc, De Boelelaan 1089a, 1081 HV Amsterdam, The Netherlands; 3 Department of Public and Occupational Health, Amsterdam UMC, Vrije Universiteit Amsterdam, Van der Boechorststraat 7, 1081 HV Amsterdam, The Netherlands; 4 Faculty of Health (Urban Vitality), Amsterdam University of Applied Sciences, Tafelbergweg 51, 1105 BD Amsterdam, The Netherlands; 5 Department of Cardiology, Amsterdam UMC, University of Amsterdam, Meibergdreef 9, 1105 AZ Amsterdam, The Netherlands; 6 Department of Epidemiology and Biostatistics, Amsterdam UMC, Vrije Universiteit Amsterdam, De Boelelaan 1089a, 1081 HV Amsterdam, The Netherlands

**Keywords:** Research misbehaviour, Research integrity, Disciplinary fields, Academic ranks, Research misconduct, Survey, Focus groups

## Abstract

**Background:**

There is increasing evidence that research misbehaviour is common, especially the minor forms. Previous studies on research misbehaviour primarily focused on biomedical and social sciences, and evidence from natural sciences and humanities is scarce. We investigated what academic researchers in Amsterdam perceived to be detrimental research misbehaviours in their respective disciplinary fields.

**Methods:**

We used an explanatory sequential mixed methods design. First, survey participants from four disciplinary fields rated perceived frequency and impact of research misbehaviours from a list of 60. We then combined these into a top five ranking of most detrimental research misbehaviours at the aggregate level, stratified by disciplinary field. Second, in focus group interviews, participants from each academic rank and disciplinary field were asked to reflect on the most relevant research misbehaviours for their disciplinary field. We used participative ranking methodology inducing participants to obtain consensus on which research misbehaviours are most detrimental.

**Results:**

In total, 1080 researchers completed the survey (response rate: 15%) and 61 participated in the focus groups (3 three to 8 eight researchers per group). Insufficient supervision consistently ranked highest in the survey regardless of disciplinary field and the focus groups confirmed this. Important themes in the focus groups were insufficient supervision, sloppy science, and sloppy peer review. Biomedical researchers and social science researchers were primarily concerned with sloppy science and insufficient supervision. Natural sciences and humanities researchers discussed sloppy reviewing and theft of ideas by reviewers, a form of plagiarism. Focus group participants further provided examples of particular research misbehaviours they were confronted with and how these impacted their work as a researcher.

**Conclusion:**

We found insufficient supervision and various forms of sloppy science to score highly on aggregate detrimental impact throughout all disciplinary fields. Researchers from the natural sciences and humanities also perceived nepotism to be of major impact on the aggregate level. The natural sciences regarded fabrication of data of major impact as well. The focus group interviews helped to understand how researchers interpreted ‘insufficient supervision’. Besides, the focus group participants added insight into sloppy science in practice. Researchers from the natural sciences and humanities added new research misbehaviours concerning their disciplinary fields to the list, such as the stealing of ideas before publication. This improves our understanding of research misbehaviour beyond the social and biomedical fields.

## Background

Most researchers think of themselves as honest and consider their work to be conducted with integrity [1–3]. In spite of this, there is increasing evidence that researchers misbehave quite frequently in their work [4–6]. Aside from the widely recognized misconducts of falsification, fabrication and plagiarism (henceforth: FFP), there is little evidence on what are perceived to be the most detrimental research misbehaviours [7–9]. Besides, it is becoming increasingly clear that research misbehaviours that may seem minor compared to FFP could have a substantial aggregate impact since they occur much more frequently than the ‘deadly sins’ [10–13].

A meta-analysis of 21 surveys investigating research misbehaviour found that about 2% of researchers admitted to falsification or fabrication. About 34% of participants admitted to questionable research practices (QRP) [4]. QRPs embody a large class of research misbehaviours, such as deleting outliers without disclosure. However, since 14 of the 21 studies included in the meta-analysis focused on biomedical researchers, it is unclear whether these proportions generalise to other disciplinary fields.

Similarly, when pooling the results of 17 studies investigating plagiarism, 1.7% of participants admitted to plagiarism [14]. However, ten of those studies used a biomedical sample. Hence, these results may not represent all sciences or the humanities. This also begs the question whether the research misbehaviours that participants were asked about were actually relevant to their own research, as some QRPs may be field or discipline-specific.

We investigated whether the research misbehaviours that are perceived detrimental vary across disciplinary fields. We distinguished four major disciplinary fields in our study: biomedical sciences, natural sciences, social sciences, and the humanities. Since FFP are relatively rare, we focus on research misbehaviours that are detrimental on the aggregate level. To get a sense of which research misbehaviours were most detrimental at the aggregate level, we also take the frequency of the research misbehaviour into account. Hence, our study aims to assess what academic researchers in Amsterdam perceive to be detrimental research misbehaviours on the aggregate level in their respective disciplinary fields.

## Methods

### Design

**Fig. 1:**
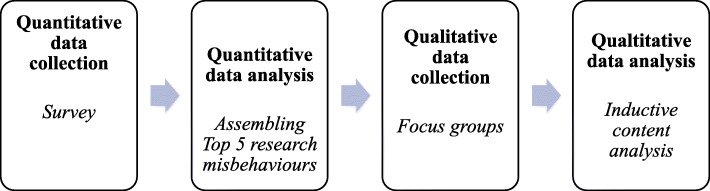
Overview of study design and analysis

We used a mixed methods sequential explanatory quantitative first design [15]. This implies that our study had two phases: (1) a quantitative phase in which we collected survey data, and (2) a qualitative phase in which we conducted focus group interviews to deepen our understanding of the survey responses (see Fig. 1).

### Ethical statement

The Scientific and Ethics Review board of the Faculty of Behavioural and Movement Sciences, Vrije Universiteit, Amsterdam reviewed and approved our study (Approval Number:VCWE-2017-017R1).

### Participants

Participants consisted of academic researchers with at least a 0.2 FTE research appointment at the Vrije Universiteit Amsterdam, University of Amsterdam or the Amsterdam University Medical Centers and included PhD students.

### Materials

We presented participants with research misbehaviours from a list of 60 major and minor misbehaviours as composed by Bouter et al. [11]. For a thorough description of the development of the list, the reader is referred to Bouter et al. [11]. The list can be found in Additional file 1.

In brief, they compiled an extensive list of over 100 research misbehaviours based on the existing literature on research misbehaviours. After removing duplications, 60 items remained which were tested for comprehensibility on 15 researchers. These 60 items were then distributed among keynote speakers and oral presenters of the World Conference on Research Integrity for review. Finally, the list of 60 was used in an invitational workshop at the 4^th^ World Conference on Research Integrity (2015) which provided final input for the phrasing of the items and the relevant response scales. The list was developed - and used by us - in English.

We used two response scales from the initial list: frequency and impact, respectively. We altered these response scales slightly by specifying the time frame or unit respondents had to keep in mind when reading the items. The impact response scale, ‘How often will this misbehaviour occur?’, was changed into (italics stress our changes): ‘How often have you observed the behaviour stated above *in the last three years*?’. This question had to be answered in reference to respondents’ main disciplinary field. Answer options were 1 (‘Never’), 2 (‘Once or twice’) and 3 (‘Three times or more’). The impact response scale, ‘If it occurs, how large will its impact be on the validity of knowledge?’, was changed into ‘If you were to observe this behaviour, how large would its impact to be on the validity of *the findings of the study at issue*?’. Responses ranged from 1 (‘Negligible’) to 5 (‘Enormous’).

### Quantitative data collection procedure

We contacted the deans and rectors from the participating institutions with a request to contact their academic researchers. The institutions shared the contact details of their researchers on the basis of a formal data sharing agreement. To explain the study’s aim, we sent all academic researchers in Amsterdam (*n* = 7548) an information letter. This letter also included a hyperlink to the privacy policy and the study protocol on our project website (see Additional files 2 and 3). One week later, we sent an invitational email to all researchers. Participants had to give informed consent and confirm that they were actually involved in research for at least 1 day per week on average (inclusion check) at the beginning of the survey. We used Qualtrics (Qualtrics, Provo, UT, USA) to build the survey.

To reduce the overall length of the survey and decrease the risk of participant fatigue [16], participants were randomly presented 20 out of 60 items from the list by Bouter et al. [11]. To preclude order effects, the order of presentation of the 20 items was also randomised.

The survey ended with three demographic items: participants’ academic rank (PhD student, postdoc, assistant professor, associate professor or full professor), disciplinary field (biomedicine, natural sciences, social sciences, and humanities) and gender (male or female). In the remainder of this paper, we distinguish three main groups of academic ranks: PhD students; postdocs and assistant professors; and associate and full professors.

The survey consisted of three parts, one of which was the list of 60 research misbehaviours described here. The remainder comprised two instruments, one about the research climate for integrity [17] and another about the degree of perceived publication pressure [18]. The data described here extend our previous findings [17] by identifying the research misbehaviours that are perceived to impact the research climate most.

### Quantitative data analysis

We preregistered our analyses on the Open Science Framework, see https://osf.io/x6t2q/register/565fb3678c5e4a66b5582f67. Here, we explain the main analyses briefly. First, we calculated the five most frequent and five most impactful research misbehaviours per academic rank and disciplinary field. Second, although falsifying data, fabricating data or committing plagiarism are most detrimental to science, they are relatively rare and therefore it is not useful to overemphasize the importance of FFP. To get a sense of which research misbehaviours were most detrimental at the aggregate level, we followed Bouter et al. [11] and multiplied the impact score of each research misbehaviour with its perceived frequency. In particular, we use the product score (multiplication) of impact and frequency as a proxy for aggregate impact throughout this manuscript. This metric ranged from 1 (negligible impact/never observed this) to 15 (enormous impact/observed this more than three times). We present these stratified top 5 rankings of detrimental research misbehaviours on the aggregate level below.

Finally, we carried out exploratory analyses to statistically assess whether the top 5 was actually a good representation of impactful research misbehaviours at the aggregate level. These analyses were not preregistered and should be treated as exploratory. Our reasoning was as follows: if a research misbehaviour could have been on #1 on the ranking, it means the research misbehaviour has substantial impact. We thus assessed the bias-corrected bootstrapped 95% confidence intervals around the mean estimates. If there was any overlap between the confidence intervals, we concluded that this research misbehaviour could have also been ranked first. If this was the case, we adjusted the rankings. Second, we used those new rankings to inspect whether there were any differences between disciplinary fields, seeing if the confidence intervals around a mean estimates overlapped between disciplinary fields.

### Qualitative data collection

We extended the survey results with focus group interviews. Our aim was twofold. First, we wanted to know whether researchers recognised the top 5 research misbehaviours we identified based on the survey as relevant for their disciplinary field. Second, if they did not recognise (some of) the research misbehaviours, we gave participants of the focus group interviews the opportunity to present and discuss other research misbehaviours that they considered (more) relevant to their disciplinary field.

We organised focus groups with researchers from three academic ranks and four disciplinary fields. These focus groups took place at the Vrije Universiteit; therefore, we only invited researchers from the Vrije Universiteit and the Amsterdam UMC (location VUmc) as they were most conveniently located.

We recruited researchers in three ways. First, we wrote to heads of department and asked them to provide e-mail addresses of potentially interested researchers. Second, we used our network of colleagues that work on unrelated topics. Third, we randomly selected researchers from the different academic ranks and disciplinary fields and invited them via e-mail where we explained the purpose of the focus group and asked them to participate. When an invitee abstained from participation (abstaining from participation was mostly due to conflicting schedules, lack of time or other reasons), we invited a new researcher, and so on until we reached a minimum of four confirmations per focus group. Note that it could thus be the case that the focus group participants had also participated in the survey that was disseminated nine months prior to the start of the focus groups. Yet, we have no information to quantify this as we did not ask about it specifically.

In total, we conducted 12 focus group interviews between March 2018 and May 2018 with 61 researchers. To encourage participants to speak freely, the groups were homogenous for academic rank and disciplinary field (see Table 1).

**Table 1:** Overview of academic researchers from Vrije Universiteit Amsterdam and Amsterdam UMC location VUmc per focus group

Academic rank	Disciplinary field^a^			
	Biomedicine	Natural sciences	Social sciences	Humanities
PhD students	5 (5)	4 (0)^E^	4 (3)^E^	6 (5)^E^
Postdocs and assistant professors	5 (4)	3 (0)	7 (3)^E^	5 (5)^E^
Associate and full professors	4 (0)	4 (0)	4 (1)^E^	7 (3)^E^
Total	14 (9)	11 (0)	13 (7)	18 (13)

^a^In brackets is the number of female researchers

^E^ Denotes focus groups that were conducted in English

A facilitator (TH or JT) led the focus groups, accompanied by an observer who made notes and ensured audiotape recording. We constructed a topic guide to direct the focus group interviews (see Additional file 4) where we presented participants with the aggregated impact top 5 of research misbehaviours that we found in the survey among researchers from their disciplinary field. We then asked participants to add new research misbehaviours that were, in their opinion, at least as relevant to their disciplinary field. As a restriction, we asked all researchers to focus on things they had actually experienced or observed, instead of something they had only heard of or read about.

We used a participative ranking method to structure the focus group discussion about the research misbehaviours. The procedure of the participative ranking method involved three steps. First, participants were presented with the five research misbehaviours that ranked highest on aggregate impact on post-its. Second, they were asked to reflect on the relevance of these behaviours for their disciplinary field and prompted to add new behaviours that we may have missed but that participants considered more relevant for their disciplinary field. All research misbehaviours were written down on post-its. Finally, participants were asked to reach consensus over a ranking of all the research misbehaviours. For that, we had created a provisional severity continuum/scale that ranged from ‘Minor’ to ‘Major’. When participants agreed on where each post-it had to be placed on the severity scale, we ended the exercise. In total, this took between 20 and 35 min. The remaining results of the focus groups will be part of another report. For an elaborate description of participative ranking methodology, the reader is referred to the guide by Ager, Stark and Potts [19].

### Qualitative data analysis

We read the transcripts and started open coding using Atlas TI© Version 8.3.0. If the transcripts were in Dutch, we assigned English codes for consistency and translated quotations. We used inductive content analysis to analyse the transcripts as it is a good method for systematically describing and understanding complex phenomena [20] and it helps to reduce rich data to meaningful concepts that capture the phenomenon of interest [21].

The themes reported below are based on the qualitatively ranked research misbehaviours according to severity as well as the transcripts of the focus group conversations. Specific research misbehaviours, e.g. ‘reviewing without feedback, harsh reviewing, reviewers not up to scratch with developments’ were clustered into broader issues, e.g. ‘sloppy reviewing’. For issues to be identified as emerging themes, the issue had to be related to the research question that involved *research* misbehaviours. Therefore, some issues that focused on political intricacies or personal integrity were disregarded. Moreover, it should be either mentioned multiple times, or during the conversation be discussed as important and powerful.

Team members (JT, TH, GW and RP) independently identified themes and these were discussed to achieve consensus and thereby increase reliability. See Additional file 5 for our code tree. Finally, we identified appropriate quotes to illustrate each theme.

## Results

### Quantitative results

**Fig. 2:**
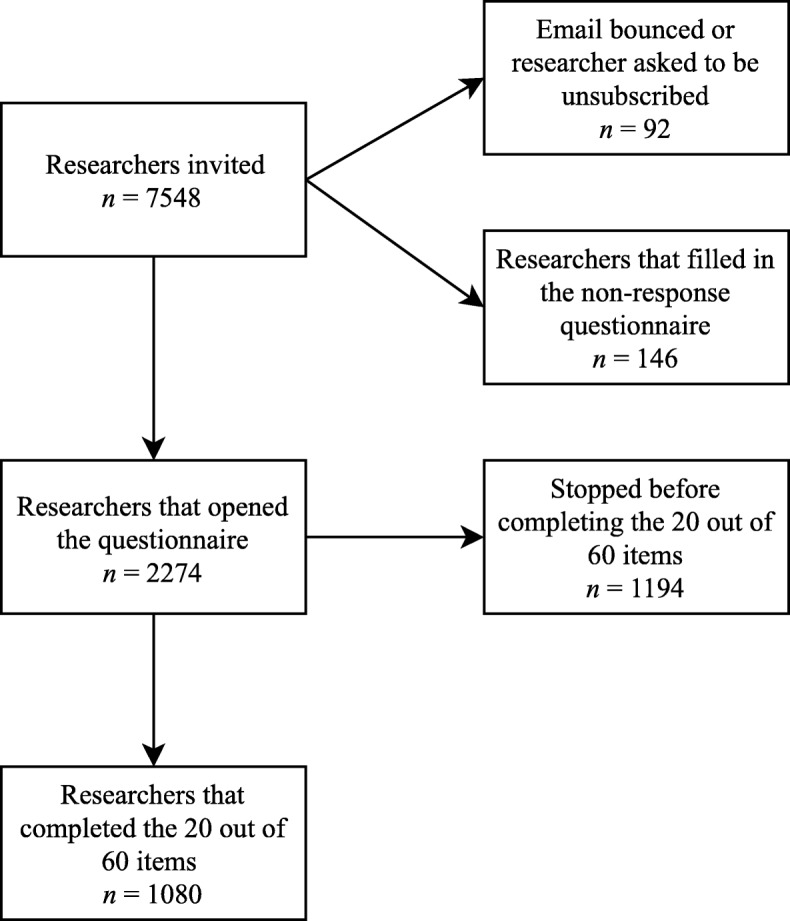
Overview of survey response rate

Ninety-two e-mail addresses were no longer in use and 146 researchers filled in the non-response questionnaire. Hence, 7310 potential respondents were left, of which 1080 researchers completed the 60 items. Survey completion rate was 15% (see Fig. 2). First, we present the quantitative top 5 of detrimental research misbehaviours on the aggregate level per disciplinary field. Second, we provide the relevant themes from the focus groups that shed more light on what these research misbehaviours mean and illustrate these with quotes.

### Disciplinary fields

A detailed description of the top 5 most frequent and most impactful research misbehaviours per disciplinary field can be found in Additional files 6 and 7. The top 5 detrimental research misbehaviours on the aggregate level stratified per rank can be found in Additional file 8. Finally, a stratified ranking of all 60 items can be found in Additional file 9.

Briefly, the misbehaviour ‘fabrication of data’ qualified as the most impactful for the validity of the study in all disciplinary fields. Most frequent research misbehaviours differed somewhat. Biomedical researchers perceived listing an author that does not qualify for authorship to be most frequent. According to natural sciences researchers and social sciences researchers*,* insufficient supervision was most frequent. Researchers in the humanities perceived selective citation to be most frequent. Humanities researchers also rated the presentation of grossly misleading information in a grant application as having major impact.

In this paper, we focus on the top 5 of most detrimental research misbehaviours on the aggregate level per disciplinary field (see Table 2).

**Table 2:** Top 5 detrimental research misbehaviours on the aggerate level by disciplinary field

Top 5 research misbehaviours per disciplinary field with *M* (*SD*)								
	Biomedicine		Natural sciences		Social sciences		Humanities	
#1	Insufficiently supervise or mentor junior co-workers	7.02 (3.63)	Insufficiently supervise or mentor junior co-workers	7.72 (4.13)	Insufficiently supervise or mentor junior co-workers	6.95 (3.78)	Insufficiently supervise or mentor junior co-workers	6.76 (3.84)
#2	Choose a clearly inadequate research design or using evidently unsuitable measurement instruments	6.04 (3.16)	Not report clearly relevant details of study methods	6.95 (3.43)	Not publish a valid ‘negative’ study	6.54 (3.98)	Use published ideas or phrases of others without referencing	6.69 (3.69)
#3	Let own convictions influence the conclusions substantially	5.99 (3.17)	Insufficiently report study flaws and limitations	6.64 (3.41)	Let own convictions influence the conclusions substantially	5.86 (2.95)	Selectively cite to enhance own findings or convictions	6.17 (3.25)
#4	Give insufficient attention to the equipment, skills or expertise which are essential to perform the study	5.64 (3.32)	Let own convictions influence the conclusions substantially	6.38 (3.27)	Choose a clearly inadequate research design or using evidently unsuitable measurement instruments	5.77 (3.38)	Choose a clearly inadequate research design or using evidently unsuitable measurement instruments	6.11 (3.37)
#5	Keep inadequate notes of the research process	5.62 (2.96)	Give insufficient attention to the equipment, skills or expertise which are essential to perform the study	6.26 (3.48)	Give insufficient attention to the equipment, skills or expertise which are essential to perform the study	5.71 (3.3)	Unfairly review papers, grant applications or colleagues applying for promotion	6.03 (4.15)

### Exploratory analyses

The following analyses were not preregistered and should be treated as exploratory.

We wanted to assess the precision of our mean estimates in Table 2. In what follows, we use bias-corrected bootstrapped 95% confidence intervals around the mean estimates.

In biomedicine, ‘Insufficient supervision’ ranked #1 and inspection of the confidence intervals indicated that no other misbehaviour could be ranked highest. There was no overlap between the confidence interval around the mean estimate for ‘Insufficient supervision’ and the confidence intervals of the research misbehaviours listed second and third. For the natural sciences, the confidence interval around ‘Insufficient supervision’ overlapped with confidence intervals up to misbehaviours ranked twelfth. The top 12 for natural sciences can be found in Additional file 10. Besides sloppy science, the top 12 for natural sciences also listed data fabrication (#7) and nepotism (#11). In the social sciences, the confidence interval around ‘Insufficient supervision’ overlapped with the confidence intervals up to the misbehaviour ranked sixth, see Additional file 10. The confidence interval around ‘Insufficient supervision’ in the humanities overlapped with research misbehaviours up to rank #12. Hence, the top 12 for the humanities can be found in Additional file 10. Besides sloppy science, the top 12 for researchers in the humanities included nepotism (#6).

To see if the updated rankings differed between disciplinary fields, we again inspected the confidence intervals around the mean estimates. Biomedical sciences perceived ‘Insufficient supervision’ to have the greatest impact on the aggregate level, but this was not different from other fields. For the natural sciences, ‘Not report clearly relevant details of study methods’ ranked second (CI 5.93–7.93). However, this rank differed significantly from the two other main disciplinary fields, i.e. the natural sciences perceived this to have a greater impact on the aggregate level than both biomedical researchers (#12, CI 4.69–5.43) and researchers in the humanities (#51, CI 2.88–3.97). In addition, insufficient attention to the expertise to perform the study (#5, CI 5.23–7.36) ranked higher on aggregate impact in for natural sciences compared to the humanities (#36, CI 3.03–4.9). Lastly, the presentation of grossly misleading information in a grant application (#9, CI 4.54–6.5) as of greater impact than researchers in the social sciences (#47, CI 3.11–4.00) and the biomedical sciences (#36, CI 3.76–4.22).

For the social sciences, not publishing a negative study ranked second (CI 5.71–7.29) and social science researchers were significantly more concerned about this than their colleagues in the humanities (#25, CI 3.5–5.00). In addition, insufficient attention to the expertise to perform the study (#5, CI 5.06–6.42) ranked higher on aggregate impact in for social sciences compared to the humanities (#36, CI 3.03–4.9). Also, ‘Reporting an unexpected finding as being hypothesised from the start’ (#6, CI 4.94–6.25) was perceived as having a greater impact on the aggregate level by social science researchers compared to researchers in the natural sciences (#34, CI 3.24–4.83) and the biomedical sciences (#17, CI 4.28–4.92).

Researchers in the humanities indicated selective citation to please editors, reviewers and colleagues (#5, CI 5.13–7.03) to have more impact on the aggregate level compared to biomedical researchers (#23, CI 4.11–4.78). Lastly, researchers in the humanities perceived the use of published ideas or phrases of others (#12) as of greater impact than biomedical researchers (#49, CI 3.29–3.85) and the natural sciences (#36, CI 3.09–5). All other comparisons between fields were nonsignificant (see Additional file 11).

### Qualitative results

From our qualitative analysis, the majority of research misbehaviours fell into one of three broad categories: issues around peer review, sloppy conduct of research and insufficient supervision. To better understand what sort of research misbehaviours researchers from a particular disciplinary field were confronted with and how these impacted their research, we zoomed in on themes that were more specific for a disciplinary field or that received more attention in their discussions. We present these themes per disciplinary field and, where possible, we identified quotes as illustrations (see Table 3 below). The rankings of research misbehaviours per focus group can be found in Additional file 12.

**Table 3:** Quotations per disciplinary field to illustrate the content of the research misbehaviour themes

Theme	Quote
Biomedicine	
Sloppy reporting	‘Take things that are reported as a decrease of 80% in tumour rate. Well, when you attempt to repeat the experiment you get a 60% decrease so obviously their 80% was the most positive result from all the times they tried…’—PhD student
Insufficient supervision	‘If you have a PhD student and you completely throw her in at the deep end, surely you increase the chance of irresponsible research’—Full professor
Inflexible reviewers	‘So everything that is novel or different, it requires an lot of effort to get that accepted in the, in the journals, due to most likely also rigid reviewers’—Assistant professor
Natural sciences	
Review misconduct	‘I had it once with a journal editor who was being really difficult about a publication of mine. And then he managed to get his own publication [with the same idea] in before mine’—Full professor
Team spirit missing	‘Research is no one man show, you have to teach them [PhD students] to also let go, it is not just theirs. The same holds for what I do, it is not just mine, it is a team effort...’—Assistant professor
Social sciences	
Sloppy reviewing	‘You’re on a grant review panel and you’re judging someone whom you have a personal or professional relationship with. You’re an editor of a journal and you don’t recuse yourself for a conflict of interest with the author of a paper’—Associate professor
Sloppy methods and statistics	‘What is so horrible about these strategies is, post-hoc storytelling, salami slicing, is how you win the game, this is how you become a professor, this what you should do. Some professors even tell you, like: this is what you should do’—Postdoctoral researcher
Insufficient supervision	‘Supervisors exploiting their PhD students. I think that can be sort of extended into any type of harassment; sexual, personal, mental harassment, whatever it is. Also about any type of power relationship that there is and… that he demands co-authorships, that supervisors say… I want to be on this paper, I am on this paper, not as a question but, you know, as a statement...’ —Postdoctoral researcher
Humanities	
Uncritical reviewing	‘What you see is that, there is no review culture, in which the standards of what constitutes good and bad publications are adequately present, to filter out actual hoaxes’—Full professor
Lack of supervision	‘I have a PhD student who got sent to me from abroad… I said well, when did you last speak to your supervisor? And he said no, no, no, because you can answer my questions better, the last couple of months I didn’t, because I was saving it up for you… While the actual supervision who will get… the credits is actually not an expert.’ —Assistant professor

Brackets added by the authors

### Biomedicine: delaying reviewers, sloppy reporting and insufficient supervision

Biomedical researchers were vexed about inflexible reviewers that either delayed the publications of their findings or that were unresponsive to valid counterarguments in rebuttal letters when the manuscript challenged the mainstream view in the field. This made it particularly hard to publish negative research findings, whereas focus group participants agreed that this was pivotal for knowledge to progress.

Another hindrance for knowledge to progress was that authors drew (wrong) conclusions based on little solid argumentation or seemed to interpret the data as it suited them. This was especially pertinent when only the most positive findings were reported, that then led to replication problems because the positive result was likely obtained by chance.

Insufficient supervision was a concern that participants recognised but they also indicated that a PhD student is expected to ask for help when in need. In addition, the supervisor can make the PhD student aware of existing time pressures, but this should be realistic and not indicate that PhD students are not allowed to take holidays. Finally, it was generally agreed upon that little supervision is not a sufficient condition for irresponsible research, yet it could increase the chances of a PhD student conduct irresponsible research.

### Natural sciences: review misconduct and no team spirit

Natural scientists brought up the topic of review misconduct. The misconduct takes the form of the editor or reviewer stealing ideas when put forth for publication or in a grant proposal. Reviewers or editors could either postpone publication and quickly publish the idea themselves or they could reject the manuscript and publish its ideas elsewhere. A similar story holds for grant proposals.

Natural science researchers also noted that insufficient communication and supervision may damage team relations and some researchers may fail to put their success into context, claiming that the success is only theirs.

### Social sciences: sloppy reviewing, sloppy design and statistics, and insufficient supervision

Social science researchers often encountered reviewers that demanded to be cited, which is obviously not the purpose of the review. Furthermore, they encountered reviewers that were not up to scratch with developments in the field. Lastly, some had experience with reviewers that failed to declare a conflict of interest as they had a previous relationship with the authors, revealing nepotism in publication review.

Another concern was the sloppy methods where researchers referred to conducting an underpowered study or failing to report a non-replication. Related was the use of ‘HARKing’ (hypothesising after results are known), where supervisors encouraged their PhD students to present an unexpected finding as being hypothesised from the start [22]. Other examples involved collecting more data when results were almost significant or just pressing PhD students into ‘finding’ an effect in the data, even when probably no actual effect was there.

Finally, concerns were voiced about insufficient supervision of PhD students. More senior researchers noted that PhD students were being held accountable for their academic projects at a very early stage in their career, when a PhD student is still learning what academic research involves. Sometimes supervisors took advantage of their PhD students, either by demanding co-authorships without a justification or by mentally intimidating their PhD students.

### Humanities: uncritical reviewing, mediocre research and scarce supervision

Uncritical reviewing was a concern of researchers from the humanities. That could involve a reviewer reviewing without specific comments, or reviewers that just accept a paper because of the authority of the author. This could be due to the fact that peer reviewing is not valued high enough by the scientific community. Another form of uncritical reviewing regarded failure to filter out fake papers that were clearly a hoax. Participants connected this to the fact that some fields lack clear publication criteria that a reviewer can use to judge a manuscript’s potential.

A second worry regarded mediocre research, which could mean research that is not value free, opaque or hastily written up, repetitive and inflating small findings. A related research misbehaviour was the stealing of original ideas from colleagues but also stealing ideas from PhD or master students and publishing it without (even) acknowledging them.

Finally, humanities researchers noted scarce supervision could lead to fraud. ‘Scarce’ could be in terms of quantity; there are very few postdocs and hence there is little day-to-day supervision of PhD students. ‘Scarce’ could also refer to the quality of supervision, such as when supervisors do not take their responsibility seriously, or when supervisors who are actually not an expert on the topic of the PhD student are assigned to be their supervisor.

## Discussion

This mixed-method study, involving a survey followed by focus groups, aimed to develop insight into what academic researchers in Amsterdam from different disciplinary fields considered to be the most detrimental research misbehaviours. There are a few important takeaways from our study. First, based on the survey results, we found insufficient supervision and various forms of sloppy science to score highly on aggregate impact throughout all disciplinary fields. Researchers from the natural sciences and humanities also perceived nepotism to be of major impact on the aggregate level. The natural sciences regarded fabrication of data of major impact as well. The focus group interviews helped us to understand how researchers interpret ‘insufficient supervision’. Besides, the focus group participants added insight into sloppy science in practice. Second, researchers from the natural sciences and humanities added new research misbehaviours concerning their disciplinary fields to the list, such as the stealing of ideas before publication. This improves our understanding of research misbehaviour, or ‘questionable research practices’ beyond the social and biomedical fields.

When comparing our findings to the literature, it is important to keep in mind that our findings are not prevalence estimates. Equating the self-reported proportion of a research misbehaviour with its prevalence has been criticised, see Fielder and Schwarz [23]. Moreover, in our survey, we asked respondents to report how often they had *witnessed* a particular research misbehaviour, not how often they had engaged in such behaviour themselves. We then combined this with the degree of impact respondents assigned to that item to obtain the ‘aggregate impact’. Because our aggregated impact metric is the product of impact (1–5) and frequency (1–3), one may wonder if we deliberately assigned impact more weight. Although this is true for the absolute score, this is not the case for the ranked aggregate impact product scores since the rank of a particular research misbehaviour does not change after recoding the impact scale.

Somewhat surprising is the consistent recognition of insufficient supervision and mentoring. We would like to reiterate that we regard insufficient supervision a research misbehaviour in itself. Like many other research misbehaviours, insufficient supervision describes non-compliance with one of the professional norms in academic research (adequate mentoring).

Yet, it seems plausible that insufficient supervision could, in some cases, lead to the supervisees unintentionally engaging in sloppy science because they were not socialised well into responsible conduct of research [24]. However, we believe that the influence of insufficient supervision may go further. If a supervisor fails to create a safe learning climate, this could lead to situations where PhD students do not feel confident to share their concerns about a mistake (e.g. in the data-analysis) or to oppose their supervisor’s interpretation. Similarly, Roberts and colleagues [25] put forth the speculation that when the supervisor creates an environment where only spectacular outcomes are valued, supervisees may engage in sloppy science because that yields the desired outcomes. Nevertheless, in our study, we did not investigate the possible reasons for research misbehaviours and investigating this would require a different research design.

The amount of literature on supervision and mentoring differs between disciplinary fields. Mentoring received extensive attention in medicine [26, 27] and substantial attention in psychology [28]. Mentoring and supervision have primarily been used as tools to foster diversity by encouraging minority groups to stay in science and engineering fields [29, 30], but received little attention in themselves. One exception is a study by Green and Bauer [31] that linked mentoring to science students’ academic success. In the humanities, mentoring was coined as a way to improve the workplace culture [32]. Interestingly, in our study, participants from the humanities expressed concerns about the lack of supervision altogether, or a supervisor who is in fact not an expert in the field. Natural sciences researchers recognised this, but added that bad mentoring or a supervisor mentoring too many PhD students can make group relations sour and ultimately slow down research.

### Strengths

Our study may be the first that investigates research misbehaviours and includes researchers from different disciplinary fields and all academic ranks. It is noteworthy that the different methods we used (quantitative survey and qualitative focus groups) led to similar results as both survey and focus group participants recognised sloppy science and insufficient supervision as relevant.

Additionally, our quantitative results largely confirm the findings by Bouter and colleagues [11]. Their population consisted of visitors of the World Conference of Research Integrity, but apparently both groups identify insufficient supervision and sloppiness as problems in contemporary academia.

### Limitations

There are some study limitations to bear in mind. We had considerable non-response. However, response bias is not a necessary consequence of a low response rate as long as the respondents are representative for the population [33]. We assessed the representativeness of our sample in two ways. First, we looked at our population that consisted of academic researchers in Amsterdam from two universities and two university medical centres. Those two university medical centres comprised 53% of the population. Biomedical researchers constituted 56% of our sample, indicating a small overrepresentation. Second, we compared our sample to national statistics on researchers in The Netherlands. As there are no national statistics on academic researchers in biomedicine, we filtered biomedical researchers out of our sample for this comparison. National statistics indicate that 32% of researchers work in the natural sciences, 41% work in social sciences and 27% in the humanities. In our sample, we find 25% of researchers to work in the natural sciences, 51% in the social sciences and 23% in the humanities. This indicates a moderate overrepresentation of the social sciences researchers and a slight underrepresentation of researchers in the natural sciences and humanities.

In addition, a high number of respondents that started answering the survey questions stopped before completing the 20 items. Before respondents were presented a random selection of 20 randomized items, they completed the Survey of Organisational Research Climate (henceforth: SOuRCe [34]). The number of participants that ‘started’ the survey included all researchers that opened the survey, even those who decided not to participate. In total, 18% of our invitees completed the SOuRCe and the later dropout rate of 3% during a survey questionnaire lies within the normal range [35].

A further limitation is that we presented participants with a random selection of 20 research misbehaviours because we feared that presenting them the full list of 60 would be too time-consuming. This type of design is sometimes called missingness by design, as all participants have missing values for some items. Based on similar surveys in the field, we estimated our response rate to be at least 15%. Since our population consisted of 7548 researchers, 15% of them answering one-third of our items would mean at least 300 responses per item. Initially, we expected more than 300 responses would be sufficient to compute reliable standard deviations, standard errors and confidence intervals.

Unfortunately, a quick glance at the width of the standard deviations in Table 2 revealed that the distribution of our scores was not normal. In fact, more than 90% of the aggregated impact variables have a skewed distribution. Consequently, we must be careful in the interpretation of the top 5. The ranking is purely based on point estimates. In fact, labelling the ranking as a top 5 may be dangerous as ‘top’ suggest that the #1 misbehaviour ranks absolutely higher than #2. Based on our explorative analyses, it can be concluded that this only holds for biomedicine (see Additional file 11). The top 5 presented in Table 2 simply lists five research misbehaviours that were impactful on the aggregate level and one should not overinterpret differences in the places on the list.

Another limitation regards the interpretation of aggregate impact. Participants did not rate the research misbehaviours to have major impact on the aggregate level, but we used the product of the perceived frequency of a research misbehaviour and the potential impact on the validity as a proxy for aggregate impact. Hence, we labelled these scores as ‘aggregate impact’ scores. The validity of this metric has no exact (mathematical) justification but is intuitively similar to e.g. the well-known QALY (Quality-Adjusted LifeYear) metric, which multiplies the subjective quality score of a state of living by the time spent in that state [36]. In the focus groups, we explicitly asked whether research misbehaviours had actual impact. As the focus groups in general confirmed the results of the survey, our notion of ‘aggregate impact’ is supported by the qualitative findings.

Furthermore, since the list of 60 research misbehaviours is not formally validated, it remains possible that survey items were unclear to participants. Nevertheless, the list was tried out at length through workshops and other types of informal review. Yet, especially researchers from the natural sciences and humanities mentioned research misbehaviours that seemed missing or at least substantially different from the list of 60, such as referees or editors that abuse their power to steal original ideas. Properly assessing the relevance of these new items would require translating the qualitative data into items and a representative sample from all disciplinary fields. To facilitate such an attempt, we provide an updated list of research misbehaviours (Additional file 13) in which items are reformulated, included as explanatory examples or added as new research misbehaviours. Validation of such a list could be an avenue for further research.

Finally, note that we explicitly asked respondents to focus on research misbehaviours that they had witnessed themselves, so this could decrease the generalisability of our findings so that they might not even apply to the population of academics in Amsterdam. Nevertheless, since sloppy science and insufficient supervision were recognised by academic researchers across disciplinary fields, it seems plausible that these research misbehaviours concern researchers outside Amsterdam as well.

### Implications

Since we found insufficient supervision to be recognised across fields, it may be worth exploring interventions that foster responsible supervision and mentoring. The connection between mentoring and responsible research may seem novel. Yet, Whitbeck [37] described an innovative type of group mentoring created to strengthen supervisors in discussing research integrity and to support research groups in comprehending the variety of integrity challenging situations they may encounter. More recently, Kalichman and Plemmons [38, 39] described a workshop curriculum for supervisors and faculty to convey responsible research in the actual research environment. Training programs like these are a step forward in making responsible supervision the norm.

## Conclusion

We found insufficient supervision and various forms of sloppy science to score highly on aggregate impact across disciplinary fields. Researchers from the natural sciences and humanities also perceived nepotism to be of major impact on the aggregate level. The natural sciences regarded fabrication of data of major impact as well. The focus group interviews helped us to understand how researchers interpreted ‘insufficient supervision’. Researchers from the natural sciences and humanities added new research misbehaviours concerning their disciplinary fields to the list, such as the stealing of ideas before publication. This improves our understanding of research misbehaviour beyond the social and biomedical fields.

## Supplementary information

**Additional file 1.** Full list of 60 items.

**Additional file 2.** Privacy policy.

**Additional file 3.** Research protocol.

**Additional file 4.** Focus group topic guide.

**Additional file 5.** Code tree. Focus group themes are colour coded where pink is social sciences, purple is humanities, blue is natural sciences and green is biomedical sciences. Higher order themes are dark purple and connect to the overall theme, which is light green. Lines signify relations (e.g. is one example of/is part of).

**Additional file 6.** Top 5 most frequent research misbehaviours by disciplinary field and academic rank. M = mean score per subgroup, SD = standard deviation. The frequency response scale ranged from 1 (‘never’), 2 (‘once or twice’) to 3 (‘three times or more’). A mean value of 2.03 (“Insufficiently mentor or supervise junior co-workers”) thus means that on average, our respondents stated seeing this research misbehaviour once or twice within the last three years.

**Additional file 7.** Top 5 most impactful research misbehaviours by disciplinary field and academic rank. M = mean score per subgroup, SD = standard deviation. Impact was scored on a 1 = ‘negligible’ and 5 = ‘major’ impact scale. The higher the mean score, the more impact the misbehaviour was perceived to have on the validity of the study’s findings.

**Additional file 8.** Top 5 most detrimental research misbehaviours on the aggregate level by academic rank. M = mean score per subgroup, SD = standard deviation. Detrimental impact on the aggregate level was computed as the product score of frequency (1-3) and impact (1-5) and thus ranged from 1 to 15. The higher the mean score, the higher the perceived aggregate impact.

**Additional file 9. **60 items ranked for aggregate impact per disciplinary field and academic rank. *M* = mean score per subgroup, *SD* = standard deviation, *N* = number of responses. PD & Asis Prof = Postdocs and assistant professors. Asso & Full Prof = Associate and full professors. Biomed = biomedical researchers, Nat = natural sciences researchers, Soc = social sciences researchers and Hum = humanities. Note that we used a missingness-by-design, so the actual number of respondents from that particular subgroup should be multiplied by three. aggregate impact was computed as the product score of frequency (1-3) and impact (1-5) and thus ranged from 1 to 15. The top 5 research misbehaviours on the aggregate level are printed in bold.

**Additional file 10.** Adjusted rankings for most detrimental research misbehaviours on the aggregate level by disciplinary field.

**Additional file 11.** Mean estimates for most detrimental research misbehaviours on the aggregate level with bootstrapped 95% confidence intervals by disciplinary field.

**Additional file 12.** Qualitative rankings of research misbehaviours according to the focus group participants. * Italics denote research misbehaviours that came from the quantitative survey. Focus groups were conducted over 3 academic ranks (see rows, PhD students, postdocs and assistant professors and associate and full professors) and 4 academic ranks (see columns, biomedical sciences, natural sciences, social sciences and humanities). The higher the misbehaviour in the rows; the more detrimental focus group participants indicated this research misbehaviour to be.

**Additional file 13. **List of 60 misbehaviours with alternative formulations and newly added items from the focus group interviews printed in *italics.* Newly added and preferred formulations of existing items are printed italic-bold and the former formulation is crossed out.

## Data Availability

The quantitative datasets generated and/or analysed during the current study are not publicly available due participants’ privacy but are available in pseudo-anonymised version from the corresponding author on reasonable request under a data sharing agreement. Transcripts of the interviews will not be provided to third parties.

## Abbreviations

FFP: Falsification, fabrication and plagiarism
FTE: Full time employment
OSF: Open science framework
QRP: Questionable research practices
