# Supplementary material for: Researchers’ perceptions of research misbehaviours: a mixed methods study among academic researchers in Amsterdam
Source: Res Integr Peer Rev. 2019 Dec 2;4:25. doi: 10.1186/s41073-019-0081-7 (PMC6886174; doi:10.1186/s41073-019-0081-7)
Supplement: Supplementary file 1 — Additional file 1. Full list of 60 items. [file 41073_2019_81_MOESM1_ESM.pdf]

Additional file 1. List of 60 misbehaviours in survey per category and in no particular order, taken from (1).

Study design
1. Propose study questions which are clearly irrelevant
2. Choose a clearly inadequate research design or using evidently unsuitable measurement instruments
3. Present grossly misleading information in a grant application
4. Write no or a clearly inadequate research protocol
5. Ignore substantial safety risks of the study to participants, workers or environment
6. Ignore substantial risks of the expected findings for society or environment
7. Importantly change the research design during the study without disclosure
8. Give insufficient attention to the equipment, skills or expertise which are essential to perform the study
**Data collection**
9. Collect more data after noticing that the results are almost statistically significant
10. Fabricate data
11. Stop data collection earlier than planned because the results are already statistically significant
12. Not adhere to pertinent laws and regulations
13. Inadequately handle or store data or materials
14. Keep inadequate notes of the research process
15. Ignore basic principles of quality assurance
**Reporting**
16. Report on data-driven hypotheses without disclosure
17. Delete data before performing data analysis without disclosure
18. Selectively delete data, modify data or add fabricated data after performing initial data-analyses
19. Perform data-analyses not stated in the study protocol without disclosure
20. Report an incorrect downwardly rounded p-value
21. Not report all study protocol-stipulated results
22. Not publish a valid ‘negative’ study
23. Report an unexpected finding as having been hypothesized from the start
24. Conceal results that contradict earlier findings or convictions

25. Not report clearly relevant details of study methods
26. Not report replication problems
27. Selectively cite to enhance own findings or convictions
28. Selectively cite to please editors, reviewers or colleagues
29. Selectively cite or cite own work to improve citation metrics
30. Let own convictions influence the conclusions substantially
31. Insufficiently report study flaws and limitations
32. Spread study results over more papers than needed
33. Duplicate publication without disclosure
34. Re-use parts of own publications without referencing
35. Re-use of previously published data without disclosure
36. Modify the results or conclusions of a study due to pressure of a sponsor
37. Failure to disclose a sponsor of the study
38. Failure to disclose a relevant financial or intellectual conflict of interest
39. Handle existing conflicts of interest inadequately
40. Communicate results to the general public before a peer reviewed publication is available
41. Deliberately communicate findings inaccurately in the media or during presentations
42. Make no clear distinction between personal views and professional comments

Collaboration

43. Take no full responsibility for the integrity of the research project and its reports
44. Refuse to share data with bona fide colleagues
45. Turn a blind eye to putative breaches of research integrity by others
46. Refuse to respond to an allegation of a breach of research integrity
47. Use unpublished ideas or phrases of others without their permission
48. Use published ideas or phrases of others without referencing
49. Unfairly review papers, grant applications or colleagues applying for promotion
50. Review one's own submitted manuscripts
51. Demand, accept or offer substantial gifts for doing a favour
52. Insufficiently supervise or mentor junior coworkers
53. Gross unfairness to collaborators
54. Add an author who doesn’t qualify for authorship
55. Demand or accept an authorship without significant contribution

56. Omit a contributor who deserves authorship
57. Not acknowledge contributors who do not qualify for authorship
58. Not ask permission from contributors for the wording of the acknowledgement
59. Not share reviewers’ comments with all co-authors
60. Submit or resubmit a paper or grant application without consent from all authors
