# Supplementary material for: Researchers’ perceptions of research misbehaviours: a mixed methods study among academic researchers in Amsterdam
Source: Res Integr Peer Rev. 2019 Dec 2;4:25. doi: 10.1186/s41073-019-0081-7 (PMC6886174; doi:10.1186/s41073-019-0081-7)
Supplement: Supplementary file 2 — Additional file 2. Privacy policy. [file 41073_2019_81_MOESM2_ESM.pdf]

Additional file 2. Privacy Policy Academic Research Climate Amsterdam

Privacy Policy - Project Academic Research Climate in Amsterdam (ARCA)

Introduction

This privacy policy informs participants how we comply with the privacy regulations of the participating institutions. We have received your email address from the Executive board of one of the four academic institutions in Amsterdam (VU, VU University Medical Center, University of Amsterdam and Academic Medical Center). With the boards of these institutions, we have made a data transfer agreement to obtain the email addresses of all active scientists. In this agreement we are committed to the privacy laws of the four institutions. The agreement stipulates that we will code all data after sampling and anonymize all obtained data before analysis. The institutions will have no access to the research data. They will only be informed with aggregated reports on a faculty level as specified below.

Aim of the study

The goal of the ARCA project is to map the research climate within the four academic institutions in Amsterdam.

Ethical considerations

The ARCA processes, manages and secures personal information with the utmost care. We work according to the Dutch Code of Conduct for Scientific Practice.

Therewith we comply with all the requirements of the Dutch Personal Data Protection Act. The study protocols were reviewed by the Medical Ethics Committee (METc) of the VU University Medical Center. The METc reviewed the protocol and stated that the study does not fall under the scope of the Medical Research Involving Human Subjects Act (WMO). The study protocol was reviewed and has been approved by the Ethics committee of the VU, faculty of Behavioral and Movement Sciences.

Scope of this Privacy Policy

This privacy policy applies to all personal information we collect as part of the ARCA project. After data collection, we will de-couple email address from responses after which email addresses will be deleted from the database before analysis and aggregation of data. Only data on age categories (by ten years), academic rank, gender and disciplinary field (fields are categorized into four types: biomedicine, social sciences, physics/engineering and humanities) will be used. These procedures

described here will effectively anonymize the data to assure that it will be practically impossible to trace back personal responses to specific questions.

Provision of anonymized data to third parties

The de-identified data (raw data after removal of email address) will not be provided to third parties unless:

• This is necessary for the purpose for which the data were obtained (i.e. submission of the de-identified dataset as part of publication policies).

• When our anonymized data is needed for research purposes in the future (i.e. a collaboration with another institution), your identity will remain protected and data will only be available after a data transfer agreement is signed by the requesting party and the researchers.

• When such a request for the anonymized data is issued, data will only be shared if the main research team reaches consensus that the requester's use of the data for future research is strictly necessary.

• If any other institution or journal wishes to us to make our data available, this will only be done via a data-transfer agreement.

Data protection

We use security and safety procedures against theft, loss and abuse by third parties and ensure that only authorized persons have access to the database. Authorized persons are the members of the research team. The research team comprises the principal investigators (J. Tijdink, MD PhD, Prof. L.M. Bouter PhD, and T.L. Haven, MSc) and the statistician of the VU / VUmc.

Data storage and location

We will store your data for a maximum of fifteen years. Data for scientific research is stored according to the legal timeframe of data storage. The data is collected through Qualtrics, which means that the data is stored on an European server.

Dissemination of results for research

We will analyze and publish the results of the survey for research purposes. Therefore we will not include data on faculty, institution or university but will only stratify the data for disciplinary fields and academic ranks.

Dissemination of results for institutions

The reports for the four institutions will be aggregated at faculty level. Because universities are split into different faculties, they will receive reports on a faculty level. Medical centers are split into research institutes and will receive one report per institute. Summaries are only written if more than 25 active scientists filled in the survey and are working at the faculty. This will make it practically impossible to identify individual participants.

Questions

If you have any questions, please send an email to Tamarinde Haven, department of philosophy via info@amsterdamresearchclimate.nl. Your query will be dealt with as quickly as possible and will remain confidential.

Withdrawing from the study

If you would like to withdraw from the study, please send an email to Tamarinde Haven, department of philosophy via info@amsterdamresearchclimate.nl to complete withdrawal.
