# Supplementary material for: Researchers’ perceptions of research misbehaviours: a mixed methods study among academic researchers in Amsterdam
Source: Res Integr Peer Rev. 2019 Dec 2;4:25. doi: 10.1186/s41073-019-0081-7 (PMC6886174; doi:10.1186/s41073-019-0081-7)
Supplement: Supplementary file 4 — Additional file 4. Focus group topic guide. [file 41073_2019_81_MOESM4_ESM.pdf]

Additional file 4. Focus groups protocol

Introduction (10 min)

- Thank the focus group participants for agreeing to the focus group
- Introduce focus group leader and observant as well as participants
- Check informed consent, if it is ok to record the interview, privacy policy and anonymity
- Explain what will happen with the results
- Explanation of how a focus group works, what are the rules for group discussions, etc.
- Ask participants if they have any questions before the focus group begins
- Brief introduction of this research project (refresh)

Interview topics (3) to be covered during the focus group (80 min)

1. Interactive assignment (think, pair, share). Assessment of the question: "What do you envision when thinking of a responsible research climate?" (25 min)
   Asks participants to individually write down three characteristics of responsible research climate (5 min). Participants are then invited to exchange these with their neighbours (5 min). Finally, groups are asked to share their characteristics with the group and discuss them deeper (10- 15 min)

2. Interactive assignment using post-its to identify misbehaviours (25 min). Participants are presented with specific misbehaviours we found important in their field and consequently invited to think of misbehaviours in their own research climate in their specific field and write down about 3-5 ones that play a big role. Small groups present their misbehaviours. (10 min)
   Verification of our findings that regard the research climate for this specific group (field X with rank Y) by means of providing them with the 5 misbehaviours. They are then asked to rank these behaviours by means of severity as a group using a major-minor scale. (5 min)
   The group as a whole is asked order these items on the major-minor scale including the new misbehaviours (10 min).

   Optional short break (10 min)

3. Group discussion: “Which barriers do you perceive in the research climate for responsible conduct of research?” (20 min)

4. How would you tackle these barriers? (10 min)
   I (see above)
   II
   II etc.

Ending the interview (10 min)

- Is there anything else you’d like to say?
- What is the main message for us to take away?
- Can we contact you in case we need any additional information of if something is unclear?
- What would you like to see coming out of our study?
- Report of this meeting with the option to comment and suggest corrections and mention the summary that will be sent to all participants to comment (member-check)
- Close the interview and thank the interviewees for their participation.
