# Supplementary material for: Researchers’ perceptions of research misbehaviours: a mixed methods study among academic researchers in Amsterdam
Source: Res Integr Peer Rev. 2019 Dec 2;4:25. doi: 10.1186/s41073-019-0081-7 (PMC6886174; doi:10.1186/s41073-019-0081-7)

Additional file 5. Code tree

Sub themes are colour coded for disciplinary field where green = biomedical sciences, blue = natural sciences, pink = social sciences and purple = humanities. Clustering themes are dark blue and the overall theme is light green. Lines signify relations (i.e. is one example of/is part of/is cause of).

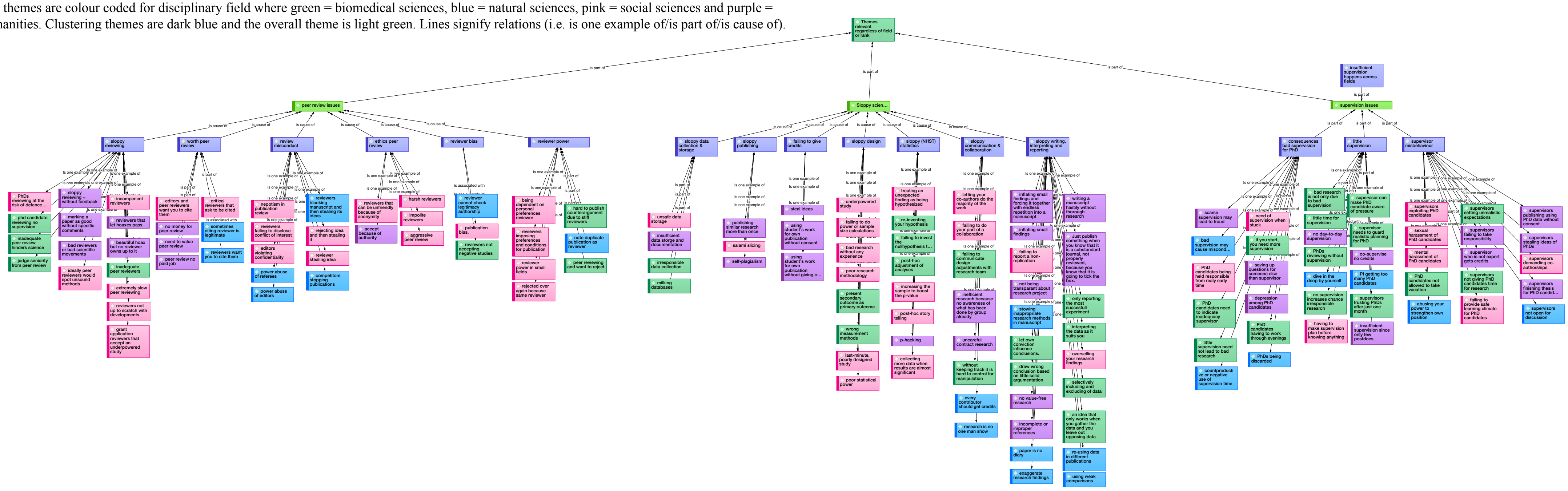

Supplement: Supplementary file 5 — Additional file 5. Code tree. Focus group themes are colour coded where pink is social sciences, purple is humanities, blue is natural sciences and green is biomedical sciences. Higher order themes are dark purple and connect to the overall theme, which is light green. Lines signify relations (e.g. is one example of/is part of). [file 41073_2019_81_MOESM5_ESM.pdf]
