# Supplementary material for: Researchers’ perceptions of research misbehaviours: a mixed methods study among academic researchers in Amsterdam
Source: Res Integr Peer Rev. 2019 Dec 2;4:25. doi: 10.1186/s41073-019-0081-7 (PMC6886174; doi:10.1186/s41073-019-0081-7)
Supplement: Supplementary file 6 — Additional file 6. Top 5 most frequent research misbehaviours by disciplinary field and academic rank. M = mean score per subgroup, SD = standard deviation. The frequency response scale ranged from 1 (‘never’), 2 (‘once or twice’) to 3 (‘three times or more’). A mean value of 2.03 (“Insufficiently mentor or supervise junior co-workers”) thus means that on average, our respondents stated seeing this research misbehaviour once or twice within the last three years. [file 41073_2019_81_MOESM6_ESM.pdf]

**Additional file 6.** Top 5 most frequent research misbehaviours by academic rank and disciplinary field.

| **Top 5** | **Academic rank, *M* (*SD*)** | | | | | | **Disciplinary field, *M* (*SD*)** | | | | | | | |
|---|---|---|---|---|---|---|---|---|---|---|---|---|---|---|
| | **PhD students** | | **Postdocs & assistant professors** | | **Associate & full professors** | | **Biomedicine** | | **Natural sciences** | | **Social sciences** | | **Humanities** | |
| **#1** | Insufficiently mentor or supervise junior co-workers | 2.03 (.77) | Demand or accept an authorship without significant contribution | 2.15 (.76) | Selectively cite to please editors, reviewers or colleagues | 2.19 (.70) | Add an author who doesn't qualify for authorship | 2.14 (.71) | Insufficiently supervise or mentor junior co-workers | 2.19 (.78) | Insufficiently supervise or mentor junior co-workers | 2.04 (.82) | Selectively cite to please editors, reviewers or colleagues | 2.03 (.81) |
| **#2** | Demand or accept an authorship without significant contribution | 2.03 (.77) | Insufficiently supervise or mentor junior co-workers | 2.1 (.75) | Selectively cite to enhance own findings or convictions | 2.09 (.70) | Demand or accept an authorship without significant contribution | 2.14 (.74) | Selectively cite to please editors, reviewers or colleagues | 2.16 (.71) | Demand or accept an authorship without significant contribution | 2 (.78) | Let own convictions influence the conclusions substantially | 2.03 (.90) |
| **#3** | Add an author who doesn't qualify for authorship | 1.97 (.73) | Selectively cite to please editors, reviewers or colleagues | 2.09 (.77) | Demand or accept an authorship without significant contribution | 2.09 (.72) | Insufficiently supervise or mentor junior co-workers | 2.06 (.72) | Not report clearly relevant details of study methods | 1.98 (.70) | Selectively cite to enhance own findings or convictions | 1.96 (.75) | Demand or accept an authorship without significant contribution | 2.03 (.74) |
| **#4** | Keep inadequate notes of the research process | 1.95 (.73) | Selectively cite or cite own work to improve citation metrics | 2.04 (.75) | Selectively cite or cite own work to improve citation metrics | 2.07 (.77) | Keep inadequate notes of the research process | 1.93 (.71) | Keep inadequate notes of the research process | 1.95 (.78) | Selectively cite to please editors, reviewers or colleagues | 1.95 (.84) | Insufficiently supervise or mentor junior co-workers | 2.02 (.77) |
| **#5** | Selectively cite to please editors, reviewers or colleagues | 1.78 (.75) | Add an author who doesn't qualify for authorship | 1.98 (.78) | Insufficiently supervise or mentor junior co-workers | 2.04 (.75) | Selectively cite to please editors, reviewers or colleagues | 1.88 (.74) | Demand or accept an authorship without significant contribution | 1.95 (.84) | Communicate results to the general public before a peer reviewed publication is available | 1.88 (.81) | Selectively cite to enhance own findings or convictions | 2.00 (.74) |

*M* = mean score per subgroup, *SD* = standard deviation. The frequency response scale ranged from 1 (‘never’), 2 (‘once or twice’) to 3 (‘three times or more’). A mean value of 2.03 (“Insufficiently mentor or supervise junior co-workers”) thus means that on average, our respondents stated seeing this research misbehaviour once or twice within the last three years.
