# Supplementary material for: Researchers’ perceptions of research misbehaviours: a mixed methods study among academic researchers in Amsterdam
Source: Res Integr Peer Rev. 2019 Dec 2;4:25. doi: 10.1186/s41073-019-0081-7 (PMC6886174; doi:10.1186/s41073-019-0081-7)
Supplement: Supplementary file 7 — Additional file 7. Top 5 most impactful research misbehaviours by disciplinary field and academic rank. M = mean score per subgroup, SD = standard deviation. Impact was scored on a 1 = ‘negligible’ and 5 = ‘major’ impact scale. The higher the mean score, the more impact the misbehaviour was perceived to have on the validity of the study’s findings. [file 41073_2019_81_MOESM7_ESM.pdf]

Additional file 7. Top 5 most impactful research misbehaviours by academic rank and disciplinary field.

Top 5	Academic rank, *M* (*SD*)						Disciplinary field, *M* (*SD*)							
	**PhD students**		**Postdocs & assistant professors**		**Associate & full professors**		**Biomedicine**		**Natural sciences**		**Social sciences**		**Humanities**	
**#1**	Fabricate data	4.55 (1.10)	Fabricate data	4.70 (.84)	Fabricate data	4.23 (1.3)	Fabricate data	4.51 (1.09)	Fabricate data	4.53 (1.28)	Fabricate data	4.62 (.94)	Fabricate data	4.44 (1.16)
**#2**	Modify the results or conclusions of a study due to pressure of a sponsor	4.41 (.94)	Selectively delete data, modify data or add fabricated data after performing initial data-analyses	4.4 (.96)	Selectively delete data, modify data or add fabricated data after performing initial data-analyses	4.22 (1.21)	Modify the results or conclusions of a study due to pressure of a sponsor	4.15 (1.16)	Modify the results or conclusions of a study due to pressure of a sponsor	4.33 (1.06)	Selectively delete data, modify data or add fabricated data after performing initial data-analyses	4.49 (.93)	Modify the results or conclusions of a study due to pressure of a sponsor	4.15 (1.11)
**#3**	Selectively delete data, modify data or add fabricated data after performing initial data-analyses	4.05 (1.32)	Modify the results or conclusions of a study due to pressure of a sponsor	4.22 (1)	Conceal results that contradict earlier findings or convictions	4.03 (.89)	Selectively delete data, modify data or add fabricated data after performing initial data-analyses	4.13 (1.21)	Selectively delete data, modify data or add fabricated data after performing initial data-analyses	4.17 (1.28)	Modify the results or conclusions of a study due to pressure of a sponsor	4.48 (.83)	Present grossly misleading information in a grant application	3.94 (.89)
**#4**	Conceal results that contradict earlier findings or convictions	3.87 (1.06)	Review one's own submitted manuscript	4.09 (1.08)	Choose a clearly inadequate research design or using evidently unsuitable measurement instruments	3.9 (1)	Review one's own submitted manuscript	3.92 (1.17)	Refuse to respond to an allegation of a breach of research integrity	3.92 (.96)	Modify the results or conclusions of a study due to pressure of a sponsor	3.99 (.96)	Selectively delete data, modify data or add fabricated data after performing initial data-analyses	3.94 (1.35)
**#5**	Choose a clearly inadequate research design or using	3.85 (1.18)	Choose a clearly inadequate research design or using	3.88 (.96)	Modify the results or conclusions of a study due to	3.88 (1.46)	Choose a clearly inadequate research design or using	3.91 (1.01)	Review one's own submitted manuscript	3.8 (1.46)	Review one's own submitted manuscripts	3.95 (1.28)	Choose a clearly inadequate research design or using	3.81 (1.13)

evidently unsuitable measurement instruments	evidently unsuitable measure-ment instruments	pressure of a sponsor	evidently unsuitable measure-ment instruments			evidently unsuitable measure-ment instruments

M = mean score per subgroup, *SD* = standard deviation. Impact was scored on a 1 = ‘negligible’ and 5 = ‘major’ impact scale. The higher the mean score, the more impact the misbehaviour was perceived to have on the validity of the study’s findings.
