# Supplementary material for: Researchers’ perceptions of research misbehaviours: a mixed methods study among academic researchers in Amsterdam
Source: Res Integr Peer Rev. 2019 Dec 2;4:25. doi: 10.1186/s41073-019-0081-7 (PMC6886174; doi:10.1186/s41073-019-0081-7)
Supplement: Supplementary file 8 — Additional file 8. Top 5 most detrimental research misbehaviours on the aggregate level by academic rank. M = mean score per subgroup, SD = standard deviation. Detrimental impact on the aggregate level was computed as the product score of frequency (1-3) and impact (1-5) and thus ranged from 1 to 15. The higher the mean score, the higher the perceived aggregate impact. [file 41073_2019_81_MOESM8_ESM.pdf]

Additional file 8. Top 5 most detrimental research misbehaviours on the aggerate level by academic rank

**Top 5**						
			**Academic rank, *M* (*SD*)**			
	**PhD students**		**Postdocs & assistant professors**		**Associate & full professors**	
**#1**	Insufficiently mentor or supervise junior coworkers	7.05 (3.76)	Insufficiently supervise or mentor junior coworkers	7.55 (3.75)	Let own convictions influence the conclusions substantially	6.74 (3.29)
**#2**	Give insufficient attention to the equipment, skills or expertise which are essential to perform the study	5.9 (3.41)	Choose a clearly inadequate research design or using evidently unsuitable measurement instruments	6.23 (3.1)	Insufficiently supervise or mentor junior coworkers	6.37 (3.65)
**#3**	Let own convictions influence the conclusions substantially	5.86 (3.17)	Let own convictions influence the conclusions substantially	6.11 (3.18)	Choose a clearly inadequate research design or using evidently unsuitable measurement instruments	6 (2.85)
**#4**	Choose a clearly inadequate research design or using evidently unsuitable measurement instruments	5.7 (3.44)	Not publish a valid ‘negative’ study	6 (3.62)	Not publish a valid ‘negative’ study	5.95 (3.57)
**#5**	Inadequately handle or store data or materials	5.64 (3.13)	Insufficiently report study flaws and limitations	5.83 (3.36)	Insufficiently report study flaws and limitations	5.94 (2.75)

M = mean score per subgroup, *SD* = standard deviation. Detrimental impact on the aggregate level was computed as the product score of frequency (1-3) and impact (1-5) and thus ranged from 1 to 15. The higher the mean score, the higher the perceived aggregate impact.
