# Supplementary material for: Researchers’ perceptions of research misbehaviours: a mixed methods study among academic researchers in Amsterdam
Source: Res Integr Peer Rev. 2019 Dec 2;4:25. doi: 10.1186/s41073-019-0081-7 (PMC6886174; doi:10.1186/s41073-019-0081-7)
Supplement: Supplementary file 9 — Additional file 9. 60 items ranked for aggregate impact per disciplinary field and academic rank. M = mean score per subgroup, SD = standard deviation, N = number of responses. PD & Asis Prof = Postdocs and assistant professors. Asso & Full Prof = Associate and full professors. Biomed = biomedical researchers, Nat = natural sciences researchers, Soc = social sciences researchers and Hum = humanities. Note that we used a missingness-by-design, so the actual number of respondents from that particular subgroup should be multiplied by three. aggregate impact was computed as the product score of frequency (1-3) and impact (1-5) and thus ranged from 1 to 15. The top 5 research misbehaviours on the aggregate level are printed in bold. [file 41073_2019_81_MOESM9_ESM.pdf]

# Additional file 9. 60 items ranked for aggregate impact per disciplinary field and academic rank.

| | Total sample | | | PhD students | | | PD & Asis.Prof | | | Asso & Prof | | | Biomed | | | Nat | | | Soc | | | Hum | | |
|---|---|---|---|---|---|---|---|---|---|---|---|---|---|---|---|---|---|---|---|---|---|---|---|---|
| *Items written out* | *N* | *M* | *SD* | *N* | *M* | *SD* | *N* | *M* | *SD* | *N* | *M* | *SD* | *N* | *M* | *SD* | *N* | *M* | *SD* | *N* | *M* | *SD* | *N* | *M* | *SD* |
| Propose study questions which are clearly irrelevant | 356 | 4.3 | 2.9 | 158 | 3.9 | 2.5 | 107 | 4.3 | 2.8 | 80 | 4.8 | 3.5 | 199 | 4.1 | 2.6 | 43 | 3.9 | 2.3 | 79 | 4.5 | 3.4 | 33 | 4.6 | 3.5 |
| Choose a clearly inadequate research design or using evidently unsuitable measurement instruments | 348 | **5.9** | **3.2** | 166 | **5.7** | **3.4** | 101 | **6.2** | **3.1** | 67 | **6** | **2.8** | 192 | **6** | **3.2** | 40 | 5.5 | 3.2 | 78 | **5.8** | **3.4** | 37 | **6.1** | **3.4** |
| Present grossly misleading information in a grant application | 357 | 4.1 | 1.8 | 162 | 4 | 1.9 | 101 | 4.1 | 1.5 | 72 | 4.5 | 1.9 | 209 | 4.1 | 1.7 | 43 | 5 | 2.7 | 70 | 3.7 | 1.6 | 31 | 4.3 | 1 |
| Collect more data after noticing that the results are almost statistically significant | 351 | 4.3 | 2.4 | 158 | 4.2 | 2.3 | 112 | 4.4 | 2.7 | 68 | 4.4 | 2.3 | 195 | 4.3 | 2.3 | 37 | 4.3 | 2.7 | 79 | 4.4 | 2.6 | 39 | 3.8 | 2.2 |
| Fabricate data | 355 | 4.8 | 1.7 | 172 | 4.8 | 1.7 | 105 | 4.9 | 1.2 | 70 | 4.8 | 2.2 | 199 | 4.7 | 1.4 | 36 | 5.5 | 2.8 | 84 | 4.8 | 1.4 | 34 | 4.9 | 2.1 |
| Report on data-driven hypotheses without disclosure | 349 | 4.2 | 2.4 | 162 | 4 | 2.2 | 105 | 4.5 | 2.5 | 72 | 4.5 | 2.6 | 193 | 4.2 | 2.4 | 40 | 3.6 | 1.8 | 81 | 4.9 | 2.8 | 34 | 3.9 | 1.3 |
| Delete data before performing data analysis without disclosure | 355 | 4.3 | 2.1 | 166 | 4.3 | 2.1 | 101 | 4.4 | 2 | 77 | 4.2 | 2.2 | 198 | 4.3 | 1.8 | 33 | 4.5 | 3.5 | 79 | 4.5 | 1.9 | 42 | 3.5 | 1.9 |
| Selectively delete data, modify data or add fabricated data after performing initial data-analyses | 348 | 4.8 | 2 | 153 | 4.7 | 2.1 | 108 | 5.1 | 2 | 73 | 4.7 | 2 | 185 | 4.7 | 1.8 | 41 | 5.4 | 3.3 | 85 | 5 | 1.6 | 35 | 4.5 | 1.7 |
| Perform data-analyses not stated in the study protocol without disclosure | 358 | 4.5 | 2.6 | 166 | 4.5 | 2.7 | 106 | 4.8 | 2.8 | 70 | 4.2 | 2.3 | 199 | 4.5 | 2.6 | 37 | 3.6 | 2 | 83 | 5 | 2.8 | 36 | 3.8 | 2.6 |
| Report an incorrect downwardly rounded p-value | 361 | 3.5 | 1.7 | 169 | 3.6 | 1.6 | 105 | 3.6 | 1.8 | 72 | 3.5 | 1.8 | 210 | 3.6 | 1.7 | 39 | 3.4 | 1.6 | 77 | 3.7 | 1.6 | 33 | 3 | 1.6 |
| Not report all study protocol-stipulated results | 347 | 4.5 | 2.6 | 150 | 4.3 | 2.6 | 108 | 4.7 | 2.7 | 73 | 4.4 | 2.5 | 185 | 4.3 | 2.1 | 33 | 4.4 | 3.2 | 85 | 5 | 3.4 | 41 | 4 | 2 |
| Not publish a valid ‘negative’ study | 355 | **5.6** | **3.4** | 159 | 5.5 | 3.4 | 117 | **6** | **3.6** | 63 | 5.5 | 3.2 | 198 | 5.5 | 3.2 | 40 | 5.5 | 3.7 | 86 | **6.5** | **4** | 30 | 4.2 | 2.4 |
| Report an unexpected finding as having been hypothesized from the start | 357 | 4.7 | 2.8 | 173 | 4.7 | 2.8 | 100 | 5 | 2.8 | 67 | 4.6 | 2.7 | 190 | 4.6 | 2.5 | 39 | 4 | 2.9 | 87 | 5.6 | 3.1 | 39 | 4.2 | 2.9 |
| Conceal results that contradict earlier findings or convictions | 344 | 5.2 | 2.5 | 156 | 5.2 | 2.5 | 101 | 4.9 | 2.5 | 75 | 5.5 | 2.4 | 181 | 5.2 | 2.4 | 41 | 5 | 2.8 | 82 | 5.3 | 2.7 | 38 | 4.8 | 2.2 |
| Take no full responsibility for the integrity of the research project and its reports | 346 | 4 | 2.3 | 168 | 4 | 2.3 | 107 | 4 | 2.1 | 58 | 4.3 | 2.8 | 200 | 3.9 | 2.2 | 34 | 5.1 | 3 | 72 | 4.1 | 2.4 | 37 | 3.7 | 1.8 |
| Refuse to share data with bona fide colleagues | 354 | 4.2 | 2.7 | 165 | 4 | 2.5 | 102 | 4.7 | 3.1 | 79 | 4 | 2.3 | 190 | 4.3 | 2.5 | 42 | 4.5 | 3.5 | 87 | 4.1 | 2.6 | 34 | 3.6 | 2.3 |
| Turn a blind eye to putative breaches of research integrity by others | 352 | 4.5 | 2.4 | 164 | 4.3 | 2 | 94 | 5 | 3.1 | 75 | 4.6 | 2.5 | 203 | 4.4 | 2.2 | 35 | 4.5 | 2.4 | 85 | 4.8 | 2.8 | 27 | 4.7 | 2.9 |
| Refuse to respond to an allegation of a breach of research integrity | 356 | 3.9 | 1.5 | 170 | 3.7 | 1.4 | 106 | 4 | 1.5 | 66 | 4.1 | 1.9 | 194 | 3.6 | 1.5 | 39 | 4.2 | 1.6 | 87 | 4.2 | 1.6 | 34 | 3.9 | 1.5 |
| Use unpublished ideas or phrases of others without their permission | 352 | 3.5 | 2.2 | 160 | 3.4 | 2 | 103 | 3.8 | 2.3 | 75 | 3.5 | 2.6 | 200 | 3.5 | 2.1 | 35 | 3.5 | 1.9 | 83 | 3.4 | 2 | 32 | 4.2 | 3.5 |
| Use published ideas or phrases of others without referencing | 356 | 3.8 | 2.3 | 164 | 3.5 | 2 | 109 | 4.1 | 2.6 | 69 | 4.2 | 2.5 | 206 | 3.6 | 2 | 34 | 3.9 | 3 | 87 | 3.9 | 2.5 | 28 | 5.1 | 3 |
| Write no or a clearly inadequate research protocol | 353 | 4.9 | 2.8 | 159 | 4.9 | 2.8 | 104 | 4.8 | 2.5 | 80 | 5.3 | 3 | 208 | 5.2 | 2.7 | 44 | 5.3 | 3.3 | 70 | 4.4 | 2.6 | 29 | 4.1 | 2.3 |
| Ignore substantial safety risks of the study to participants, workers or environment | 360 | 3.6 | 1.8 | 175 | 3.7 | 1.7 | 102 | 3.7 | 1.9 | 68 | 3.2 | 2 | 210 | 3.7 | 1.8 | 42 | 3.5 | 1.7 | 74 | 3.6 | 1.7 | 33 | 2.8 | 1.9 |
| Stop data collection earlier than planned because the results are already statistically significant | 350 | 3.8 | 2 | 162 | 3.8 | 1.6 | 108 | 3.9 | 2.3 | 70 | 3.7 | 2.3 | 189 | 3.8 | 1.7 | 43 | 3.4 | 2.3 | 76 | 4 | 2.2 | 42 | 3.9 | 2.4 |
| Not adhere to pertinent laws and regulations | 350 | 3.8 | 1.7 | 170 | 3.8 | 1.7 | 87 | 3.8 | 1.6 | 79 | 3.7 | 1.9 | 184 | 3.8 | 1.9 | 37 | 3.8 | 1.9 | 91 | 3.5 | 1.5 | 37 | 4.2 | 1.6 |
| Not report clearly relevant details of study methods | 356 | 5.2 | 3 | 169 | 4.9 | 2.8 | 109 | 5.7 | 3.3 | 71 | 5.3 | 3.1 | 205 | 5.1 | 2.9 | 40 | **7** | **3.4** | 78 | 5.3 | 3 | 32 | 3.4 | 1.8 |
| Not report replication problems | 353 | 4.7 | 2.7 | 155 | 4.7 | 2.9 | 109 | 5.1 | 2.3 | 69 | 4.1 | 2.7 | 187 | 4.6 | 2.4 | 49 | 4.3 | 2.8 | 88 | 5.2 | 2.9 | 29 | 4 | 3 |
| Selectively cite to enhance own findings or convictions | 359 | 5.5 | 2.9 | 157 | 5.1 | 2.9 | 115 | 5.8 | 2.7 | 77 | **5.9** | **2.7** | 210 | 5.5 | 2.9 | 45 | 5.2 | 2.7 | 73 | 5.5 | 2.4 | 30 | **6.2** | **3.2** |
| Selectively cite to please editors, reviewers or colleagues | 344 | 4.8 | 2.8 | 161 | 4.5 | 2.7 | 108 | 5.2 | 2.8 | 62 | 4.9 | 2.9 | 192 | 4.4 | 2.4 | 45 | 5 | 3.2 | 65 | 5 | 3.1 | 39 | 6 | 3.1 |
| Selectively cite or cite own work to improve citation metrics | 344 | 4.2 | 2.5 | 177 | 3.9 | 2.4 | 100 | 4.6 | 2.7 | 54 | 4.4 | 2.8 | 203 | 4.2 | 2.5 | 34 | 3.9 | 2.8 | 73 | 4.2 | 2.2 | 32 | 4.1 | 3.3 |
| Let own convictions influence the conclusions substantially | 362 | **6.1** | **3.2** | 171 | **5.9** | **3.2** | 104 | **6.1** | **3.2** | 69 | **6.7** | **3.3** | 202 | **6** | **3.2** | 37 | **6.4** | **3.3** | 84 | **5.9** | **2.9** | 35 | **6.7** | **3.7** |
| Insufficiently report study flaws and limitations | 341 | 5.5 | 3.1 | 156 | 5.4 | 2.9 | 101 | **5.8** | **3.4** | 70 | 5.6 | 3.1 | 196 | 5.4 | 3 | 36 | **6.6** | **3.4** | 71 | 5.2 | 3.3 | 36 | 5.4 | 2.6 |
| Spread study results over more papers than needed | 353 | 4.1 | 2.6 | 160 | 3.9 | 2.6 | 104 | 4.5 | 2.7 | 72 | 4.1 | 2.4 | 205 | 4.1 | 2.6 | 36 | 4.1 | 2.3 | 74 | 4.3 | 2.7 | 34 | 4.1 | 2.7 |
| Duplicate publication without disclosure | 362 | 3.3 | 1.7 | 156 | 3.4 | 1.7 | 115 | 3.3 | 1.8 | 73 | 3.1 | 1.7 | 191 | 3.2 | 1.5 | 44 | 3.9 | 2.3 | 87 | 3.3 | 1.6 | 36 | 3.2 | 1.7 |
| Re-use parts of own publications without referencing | 354 | 3.2 | 1.8 | 173 | 2.9 | 1.7 | 94 | 3.5 | 1.9 | 76 | 3.7 | 2 | 213 | 3 | 1.8 | 37 | 3.1 | 1.5 | 70 | 3.2 | 1.8 | 33 | 4.2 | 2.4 |
| Unfairly review papers, grant applications or colleagues applying for promotion | 357 | 4.9 | 3 | 169 | 4.1 | 2.2 | 101 | 5.7 | 3.6 | 73 | **5.9** | **3.6** | 208 | 4.6 | 2.7 | 45 | 5.3 | 3.6 | 67 | 5.3 | 3 | 34 | **6** | **4.1** |
| Review one's own submitted manuscripts | 364 | 4.1 | 1.5 | 161 | 3.9 | 1.3 | 111 | 4.4 | 1.6 | 78 | 4.1 | 1.6 | 207 | 4.1 | 1.4 | 45 | 3.9 | 1.4 | 79 | 4.1 | 1.6 | 30 | 3.9 | 1.5 |
| Demand, accept or offer substantial gifts for doing a favour | 357 | 3.4 | 1.6 | 172 | 3.3 | 1.5 | 99 | 3.5 | 2 | 71 | 3.4 | 1.4 | 203 | 3.2 | 1.5 | 40 | 4.2 | 2.4 | 76 | 3.2 | 1.3 | 36 | 3.8 | 1.4 |
| Insufficiently supervise or mentor junior coworkers | 355 | **7.1** | **3.7** | 157 | **7.1** | **3.8** | 107 | **7.6** | **3.8** | 78 | **6.4** | **3.7** | 197 | **7** | **3.6** | 32 | **7.7** | **4.1** | 77 | **6.9** | **3.8** | 46 | **6.8** | **3.8** |
| Gross unfairness to collaborators | 348 | 3.9 | 2.6 | 145 | 3.7 | 2.4 | 106 | 4 | 2.8 | 81 | 4 | 2.3 | 207 | 3.9 | 2.4 | 34 | 4.5 | 2.8 | 75 | 3.7 | 2.5 | 30 | 4 | 3.3 |
| Submit or resubmit a paper or grant application without consent from all authors | 364 | 3.2 | 1.9 | 172 | 3.1 | 1.8 | 118 | 3.3 | 2.1 | 59 | 3.3 | 2.1 | 204 | 3.3 | 2 | 39 | 3.2 | 1.7 | 81 | 3 | 2 | 38 | 2.9 | 1.6 |
| Ignore substantial risks of the expected findings for society or environment | 340 | 3.7 | 1.8 | 160 | 3.8 | 1.6 | 95 | 3.6 | 1.6 | 70 | 3.9 | 2.6 | 197 | 3.8 | 1.5 | 37 | 3.4 | 1.9 | 69 | 3.7 | 2 | 33 | 3.5 | 3 |
| Importantly change the research design during the study without disclosure | 354 | 4.4 | 2.1 | 167 | 4.3 | 2.1 | 103 | 4.7 | 2.3 | 67 | 4.2 | 2 | 190 | 4.4 | 2.1 | 32 | 4.3 | 2.5 | 92 | 4.3 | 2.2 | 39 | 4.4 | 2.2 |
| Give insufficient attention to the equipment, skills or expertise which are essential to perform study | 344 | **5.6** | **3.4** | 162 | **5.9** | **3.4** | 110 | 5.6 | 3.2 | 55 | 5.1 | 3.5 | 183 | **5.6** | **3.3** | 39 | **6.3** | **3.5** | 89 | **5.7** | **3.3** | 30 | 3.9 | 2.9 |
| Inadequately handle or store data or materials | 356 | 4.6 | 2.8 | 164 | 4.9 | 3 | 102 | 4.3 | 2.6 | 76 | 4.6 | 2.8 | 207 | 4.9 | 2.8 | 38 | 4.8 | 3.2 | 72 | 4.3 | 2.9 | 38 | 3.7 | 2.5 |
| Keep inadequate notes of the research process | 357 | 5.5 | 3.1 | 167 | **5.6** | **3.1** | 105 | 5.6 | 3.2 | 73 | 5.2 | 2.8 | 200 | **5.6** | **3** | 40 | 6 | 3.7 | 81 | 5.4 | 3 | 34 | 4.5 | 3 |
| Ignore basic principles of quality assurance | 356 | 5.3 | 2.8 | 170 | 5 | 2.8 | 103 | 5.5 | 2.6 | 79 | 5.8 | 3.1 | 202 | 5.2 | 2.8 | 41 | 5.3 | 2.8 | 80 | 5.4 | 2.9 | 33 | 5.3 | 2.5 |
| Re-use of previously published data without disclosure | 352 | 3.2 | 1.7 | 174 | 3.1 | 1.4 | 84 | 3.5 | 2 | 83 | 3.3 | 2.1 | 211 | 3.1 | 1.5 | 37 | 3 | 1.6 | 71 | 3.5 | 2.3 | 32 | 3.6 | 1.7 |
| Modify the results or conclusions of a study due to pressure of a sponsor | 361 | 4.6 | 1.5 | 172 | 4.8 | 1.4 | 117 | 4.5 | 1.2 | 59 | 4.3 | 2.3 | 194 | 4.5 | 1.5 | 43 | 4.7 | 1.7 | 85 | 4.8 | 1.6 | 39 | 4.4 | 1.4 |
| Failure to disclose a sponsor of the study | 353 | 3.2 | 1.5 | 171 | 3.1 | 1.4 | 111 | 3.2 | 1.5 | 59 | 3.4 | 1.8 | 191 | 3.2 | 1.5 | 40 | 3.1 | 1.4 | 81 | 3.3 | 1.6 | 38 | 3.1 | 1.3 |
| Failure to disclose a relevant financial or intellectual conflict of interest | 358 | 3.6 | 1.8 | 167 | 3.4 | 1.5 | 97 | 3.6 | 1.9 | 78 | 3.8 | 2.4 | 197 | 3.6 | 1.8 | 35 | 3.2 | 1.9 | 81 | 4 | 2 | 45 | 4.7 | 2.9 |
| Handle existing conflicts of interest inadequately | 346 | 4.1 | 2.5 | 165 | 3.8 | 2.2 | 97 | 4.5 | 2.5 | 71 | 4.4 | 3 | 192 | 4.1 | 2.4 | 40 | 4.2 | 2.8 | 68 | 3.8 | 2.4 | 33 | 3.8 | 1.4 |
| Communicate results to the general public before a peer reviewed publication is available | 358 | 3.9 | 2.2 | 172 | 3.7 | 2 | 105 | 3.9 | 2.4 | 64 | 4.4 | 2.4 | 194 | 3.9 | 2.1 | 39 | 3.5 | 2 | 90 | 4.1 | 2.5 | 37 | 5.2 | 3.5 |
| Deliberately communicate findings inaccurately in the media or during presentations | 368 | 4.6 | 2.5 | 163 | 4.5 | 2.6 | 118 | 4.7 | 2.5 | 71 | 4.8 | 2.7 | 193 | 4.5 | 2.3 | 46 | 4.5 | 2.7 | 87 | 4.6 | 2.5 | 38 | 5.7 | 3.7 |
| Make no clear distinction between personal views and professional comments | 338 | 4.9 | 3.1 | 157 | 4.4 | 2.9 | 102 | 5.1 | 3.3 | 68 | 5.4 | 3.1 | 176 | 4.7 | 2.8 | 37 | 4.3 | 2.9 | 85 | 5.2 | 3.4 | 43 | 3.7 | 3.4 |
| Add an author who doesn’t qualify for authorship | 355 | 3.7 | 2.5 | 179 | 3.9 | 2.6 | 93 | 4 | 2.6 | 66 | 3.2 | 1.9 | 207 | 3.9 | 2.3 | 36 | 3.7 | 2.8 | 67 | 3.4 | 1.9 | 32 | 5.2 | 3.5 |
| Demand or accept an authorship without significant contribution | 346 | 4.3 | 2.8 | 162 | 4.3 | 2.9 | 95 | 4.7 | 3.1 | 78 | 3.9 | 2.3 | 197 | 4.3 | 2.8 | 43 | 3.6 | 2.6 | 73 | 4.2 | 2.8 | 38 | 3.7 | 2.2 |
| Omit a contributor who deserves authorship | 352 | 3.5 | 2.3 | 166 | 3.4 | 2.1 | 103 | 3.9 | 2.5 | 71 | 3 | 2.1 | 180 | 3.4 | 2.3 | 47 | 3.7 | 2.1 | 85 | 3.3 | 2.2 | 41 | 3 | 2.1 |
| Not acknowledge contributors who do not qualify for authorship | 342 | 2.9 | 2.2 | 163 | 2.7 | 1.9 | 101 | 3.3 | 2.3 | 69 | 3.1 | 2.8 | 191 | 2.9 | 2.2 | 38 | 3.1 | 2.5 | 72 | 3 | 2.1 | 42 | 3.2 | 1.5 |
| Not ask permission from contributors for the wording of the acknowledgement | 350 | 2.6 | 1.7 | 158 | 2.5 | 1.6 | 105 | 2.8 | 1.9 | 73 | 2.7 | 1.9 | 190 | 2.7 | 1.7 | 41 | 2.5 | 1.5 | 80 | 2.4 | 1.9 | 38 | 2.9 | 2 |
| Not share reviewers’ comments with all co-authors | 353 | 3.5 | 2 | 159 | 3.4 | 2 | 103 | 3.5 | 1.9 | 74 | 3.6 | 2.1 | 195 | 3.6 | 2.1 | 34 | 3.4 | 1.6 | 82 | 3.3 | 1.9 | 40 | 3.1 | 1 |

M = mean score per subgroup, SD = standard deviation, N = number of responses.
PD & Asis Prof = Postdocs and assistant professors. Asso & Full Prof = Associate and full professors.
Biomed = biomedical researchers, Nat = natural sciences researchers, Soc = social sciences researchers and Hum = humanities.
Note that we used a missingness-by-design, so the actual number of respondents from that particular subgroup should be multiplied by three.
aggregate impact was computed as the product score of frequency (1-3) and impact (1-5) and thus ranged from 1 to 15.
The top 5 misbehaviours are printed in bold.
