# Supplementary material for: Researchers’ perceptions of research misbehaviours: a mixed methods study among academic researchers in Amsterdam
Source: Res Integr Peer Rev. 2019 Dec 2;4:25. doi: 10.1186/s41073-019-0081-7 (PMC6886174; doi:10.1186/s41073-019-0081-7)
Supplement: Supplementary file 10 — Additional file 10. Adjusted rankings for most detrimental research misbehaviours on the aggregate level by disciplinary field. [file 41073_2019_81_MOESM10_ESM.pdf]

**Additional file 10.** Adjusted rankings for most detrimental research misbehaviours on the aggregate level by disciplinary field

| | **Biomedical researchers, bootstrapped, μ, Bca 95% CI** | **Mean** | **LB** | **UB** |
|---|---|---|---|---|
| #1 | Insufficient supervision | 7.02 | 6.53 | 7.49 |

| | **Natural sciences researchers, bootstrapped, μ, Bca 95% CI** | **Mean** | **LB** | **UB** |
|---|---|---|---|---|
| #1 | Insufficient supervision | 7.72 | 6.38 | 9.19 |
| #2 | *Not report clearly relevant details of study methods (B, H)* | 6.95 | 5.93 | 7.93 |
| #3 | Insufficiently report study flaws and limitations | 6.64 | 5.58 | 7.74 |
| #4 | Let own convictions influence the conclusions substantially | 6.38 | 5.35 | 7.35 |
| #5 | *Give insufficient attention to the equipment, skills or expertise which are essential to perform the study (* | 6.26 | 5.23 | 7.36 |
| #6 | Keep inadequate notes of the research process | 6 | 5.03 | 7.09 |
| #7 | Not publish a valid 'negative' study | 5.52 | 4.48 | 6.63 |
| #8 | Fabricate data | 5.5 | 4.69 | 6.39 |
| #9 | Choose a clearly inadequate research design or using evidently unsuitable measurement instruments | 5.45 | 4.55 | 6.4 |
| #10 | *Present grossly misleading information in a grant application (B, S)* | 5.42 | 4.54 | 6.5 |
| #11 | Selectively delete data, modify data or add fabricated data after performing initial data-analyses | 5.39 | 4.54 | 6.42 |
| #12 | Unfairly review papers, grant applications or colleagues applying for promotion | 5.31 | 4.42 | 6.38 |

| | **Social sciences researchers, bootstrapped, μ, Bca 95% CI** | **Mean** | **LB** | **UB** |
|---|---|---|---|---|
| #1 | Insufficient supervision | 6.95 | 6.13 | 7.78 |
| #2 | *Not publish a valid 'negative' study (H)* | 6.53 | 5.71 | 7.29 |
| #3 | Let own convictions influence the conclusions substantially | 5.86 | 5.24 | 6.48 |
| #4 | Choose a clearly inadequate research design or using evidently unsuitable measurement instruments | 5.77 | 5.06 | 6.51 |
| #5 | *Give insufficient attention to the equipment, skills or expertise which are essential to perform the study (* | 5.71 | 5.06 | 6.42 |
| #6 | *Report an unexpected finding as having been hypothesized from the start (B, N)* | 5.6 | 4.94 | 6.25 |

| | **Humanities researchers, bootstrapped, μ, Bca 95% CI** | **Mean** | **LB** | **UB** |
|---|---|---|---|---|
| #1 | Insufficient supervision | 6.76 | 5.7 | 7.74 |
| #2 | Let own convictions influence the conclusions substantially | 6.69 | 5.46 | 8 |
| #3 | Selectively cite to enhance own findings or convictions | 6.17 | 5.17 | 7.23 |
| #4 | Choose a clearly inadequate research design or using evidently unsuitable measurement instruments | 6.11 | 5.11 | 7.14 |
| #5 | *Selectively cite to please editors, reviewers or colleagues (B)* | 6.03 | 5.13 | 7.03 |
| #6 | Unfairly review papers, grant applications or colleagues applying for promotion | 6.03 | 4.77 | 7.41 |
| #7 | Make no clear distinction between personal views and professional comments | 5.68 | 4.57 | 6.82 |
| #8 | Insufficiently report study flaws and limitations | 5.42 | 4.64 | 6.14 |
| #9 | Ignore basic principles of quality assurance | 5.27 | 4.48 | 6.03 |
| #10 | Deliberately communicate findings inaccurately in the media or during presentations | 5.24 | 4.11 | 6.35 |
| #11 | Demand or accept an authorship without significant contribution | 5.22 | 4.22 | 6.25 |
| #12 | *Use published ideas or phrases of others without referencing (B, N)* | 5.07 | 4.07 | 6.21 |

*Italics indicate misbehaviours that were perceived significantly different on aggregate impact by researchers from this disciplinary field*
*The group for which the ranking differed is indicated beteween brackets where: B = biomedicine, N = natural sciences, S = social sciences, H = humanities*

LB = lower bound Bca bootstrapped confidence interval, UB = upper bound Bca bootstrapped confidence interval
