# Supplementary material for: Researchers’ perceptions of research misbehaviours: a mixed methods study among academic researchers in Amsterdam
Source: Res Integr Peer Rev. 2019 Dec 2;4:25. doi: 10.1186/s41073-019-0081-7 (PMC6886174; doi:10.1186/s41073-019-0081-7)
Supplement: Supplementary file 11 — Additional file 11. Mean estimates for most detrimental research misbehaviours on the aggregate level with bootstrapped 95% confidence intervals by disciplinary field. [file 41073_2019_81_MOESM11_ESM.pdf]

Additional file 11. Mean estimates for most detrimental research misbehaviours on the aggregate level with bootstrapped 95% confidence intervals by disciplinary field.

Biomedical researchers, μ, bootstrapped Bca 95% CI Lower Bound (LB), Upper Bound (UB)	μ	CI LB	CI UB
Insufficiently supervise or mentor junior coworkers	7.02	6.53	7.49
Choose a clearly inadequate research design or using evidently unsuitable measurement instruments	6.04	5.64	6.46
Let own convictions influence the conclusions substantially	5.99	5.59	6.41
Give insufficient attention to the equipment, skills or expertise which are essential to perform the study	5.64	5.21	6.1
Keep inadequate notes of the research process	5.62	5.23	6.06
Selectively cite to enhance own findings or convictions	5.52	5.1	5.94
Not publish a valid 'negative' study	5.48	5.06	5.89
Insufficiently report study flaws and limitations	5.45	5.09	5.85
Ignore basic principles of quality assurance	5.25	4.84	5.68
Conceal results that contradict earlier findings or convictions	5.23	4.91	5.56
Write no or a clearly inadequate research protocol	5.18	4.82	5.58
Not report clearly relevant details of study methods	5.07	4.69	5.43
Inadequately handle or store data or materials	4.87	4.52	5.22
Selectively delete data, modify data or add fabricated data after performing initial data-analyses	4.72	4.47	4.98
Fabricate data	4.68	4.49	4.87
Make no clear distinction between personal views and professional comments	4.68	4.29	5.07
Report an unexpected finding as having been hypothesized from the start	4.62	4.28	4.92
Not report replication problems	4.62	4.3	4.97
Unfairly review papers, grant applications or colleagues applying for promotion	4.56	4.21	4.88
Perform data-analyses not stated in the study protocol without disclosure	4.51	4.15	4.87
Modify the results or conclusions of a study due to pressure of a sponsor	4.51	4.29	4.72
Deliberately communicate findings inaccurately in the media or during presentations	4.51	4.21	4.8
Selectively cite to please editors, reviewers or colleagues	4.44	4.11	4.78
Turn a blind eye to putative breaches of research integrity by others	4.41	4.14	4.69
Importantly change the research design during the study without disclosure	4.38	4.09	4.71
Delete data before performing data analysis without disclosure	4.34	4.12	4.59
Demand or accept an authorship without significant contribution	4.34	3.96	4.72
Collect more data after noticing that the results are almost statistically significant	4.31	4.03	4.63
Not report all study protocol-stipulated results	4.3	4.04	4.57
Selectively cite or cite own work to improve citation metrics	4.2	3.88	4.53
Report on data-driven hypotheses without disclosure	4.18	3.88	4.49
Handle existing conflicts of interest inadequately	4.12	3.8	4.46
Review one's own submitted manuscripts	4.11	3.92	4.3
Propose study questions which are clearly irrelevant	4.1	3.76	4.46
Spread study results over more papers than needed	4.1	3.76	4.42
Present grossly misleading information in a grant application	3.99	3.76	4.22
Add an author who doesn't qualify for authorship	3.9	3.59	4.19
Take no full responsibility for the integrity of the research project and its reports	3.89	3.63	4.19
Communicate results to the general public before a peer reviewed publication is available	3.89	3.58	4.21
Gross unfairness to collaborators	3.85	3.53	4.19
Ignore substantial risks of the expected findings for society or environment	3.81	3.62	4
Not adhere to pertinent laws and regulations	3.77	3.51	4.03
Stop data collection earlier than planned because the results are already statistically significant	3.76	3.53	4.04
Ignore substantial safety risks of the study to participants, workers or environment	3.74	3.52	3.95
Not ask permission from contributors for the wording of the acknowledgement	3.64	3.35	3.92
Refuse to respond to an allegation of a breach of research integrity	3.63	3.42	3.83
Report an incorrect downwardly rounded p-value	3.6	3.38	3.81
Failure to disclose a relevant financial or intellectual conflict of interest	3.57	3.33	3.8
Use published ideas or phrases of others without referencing	3.56	3.29	3.85
Use unpublished ideas or phrases of others without their permission	3.54	3.29	3.82
Omit a contributor who deserves authorship	3.39	3.09	3.67
Submit or resubmit a paper or grant application without consent from all authors	3.33	3.05	3.58
Demand, accept or offer substantial gifts for doing a favour	3.22	3.03	3.4
Duplicate publication without disclosure	3.18	2.95	3.38
Failure to disclose a sponsor of the study	3.18	2.98	3.38
Re-use of previously published data without disclosure	3.07	2.87	3.27
Re-use parts of own publications without referencing	3.04	2.81	3.27
Not acknowledge contributors who do not qualify for authorship	2.88	2.62	3.18
Refuse to share data with bona fide colleagues	2.59	2.4	2.79
Not share reviewers' comments with all co-authors	2.29	3.96	4.65

Natural sciences researchers, μ, bootstrapped Bca 95% CI Lower Bound (LB), Upper Bound (UB)	μ	CI LB	CI UB
Insufficiently supervise or mentor junior coworkers	7.72	6.38	9.19
Not report clearly relevant details of study methods	6.95	5.93	7.93
Insufficiently report study flaws and limitations	6.64	5.58	7.74
Let own convictions influence the conclusions substantially	6.38	5.35	7.35
Give insufficient attention to the equipment, skills or expertise which are essential to perform the study	6.26	5.23	7.36
Keep inadequate notes of the research process	6	5.03	7.09
Not publish a valid 'negative' study	5.52	4.48	6.63
Fabricate data	5.5	4.69	6.39
Choose a clearly inadequate research design or using evidently unsuitable measurement instruments	5.45	4.55	6.4
Present grossly misleading information in a grant application	5.42	4.54	6.5
Selectively delete data, modify data or add fabricated data after performing initial data-analyses	5.39	4.54	6.42
Unfairly review papers, grant applications or colleagues applying for promotion	5.31	4.42	6.38
Ignore basic principles of quality assurance	5.29	4.54	6.17
Write no or a clearly inadequate research protocol	5.25	4.34	6.16
Selectively cite to enhance own findings or convictions	5.18	4.41	6
Take no full responsibility for the integrity of the research project and its reports	5.12	4.15	6.32
Conceal results that contradict earlier findings or convictions	4.98	4.24	5.8
Selectively cite to please editors, reviewers or colleagues	4.96	4.11	5.94
Inadequately handle or store data or materials	4.76	3.92	5.68
Modify the results or conclusions of a study due to pressure of a sponsor	4.72	4.32	5.17
Gross unfairness to collaborators	4.53	3.65	5.41
Refuse to share data with bona fide colleagues	4.5	3.52	5.63
Deliberately communicate findings inaccurately in the media or during presentations	4.5	3.84	5.26
Delete data before performing data analysis without disclosure	4.49	3.5	5.76
Turn a blind eye to putative breaches of research integrity by others	4.49	3.81	5.29
Not report all study protocol-stipulated results	4.42	3.49	5.52
Not report replication problems	4.33	3.65	5.13
Collect more data after noticing that the results are almost statistically significant	4.32	3.6	5.08
Importantly change the research design during the study without disclosure	4.31	3.59	5.15
Make no clear distinction between personal views and professional comments	4.27	3.43	5.19
Handle existing conflicts of interest inadequately	4.23	3.47	5.1
Refuse to respond to an allegation of a breach of research integrity	4.21	3.82	4.62
Demand, accept or offer substantial gifts for doing a favour	4.15	3.55	4.8
Spread study results over more papers than needed	4.08	3.42	4.78
Report an unexpected finding as having been hypothesized from the start	3.97	3.23	4.83
Use published ideas or phrases of others without referencing	3.94	3.09	5
Selectively cite or cite own work to improve citation metrics	3.94	3.15	4.87
Propose study questions which are clearly irrelevant	3.93	3.3	4.56
Duplicate publication without disclosure	3.89	3.32	4.5
Review one's own submitted manuscripts	3.87	3.47	4.24
Not adhere to pertinent laws and regulations	3.81	3.27	3.35
Add an author who doesn't qualify for authorship	3.72	2.97	4.55
Omit a contributor who deserves authorship	3.72	3.17	4.3
Report on data-driven hypotheses without disclosure	3.63	2.13	4.2
Demand or accept an authorship without significant contribution	3.61	2.91	4.37
Perform data-analyses not stated in the study protocol without disclosure	3.57	3.05	4.08
Communicate results to the general public before a peer reviewed publication is available	3.49	2.95	4.1
Use unpublished ideas or phrases of others without their permission	3.46	2.89	4.09
Ignore substantial safety risks of the study to participants, workers or environment	3.45	3	3.91
Not ask permission from contributors for the wording of the acknowledgement	3.44	2.94	3.94
Report an incorrect downwardly rounded p-value	3.41	2.95	3.87
Stop data collection earlier than planned because the results are already statistically significant	3.4	2.84	4.12
Ignore substantial risks of the expected findings for society or environment	3.35	2.76	3.92
Failure to disclose a relevant financial or intellectual conflict of interest	3.2	2.63	3.79
Submit or resubmit a paper or grant application without consent from all authors	3.18	2.69	3.69
Re-use parts of own publications without referencing	3.14	2.7	3.6
Not acknowledge contributors who do not qualify for authorship	3.11	2.47	3.82
Failure to disclose a sponsor of the study	3.1	2.7	3.45
Re-use of previously published data without disclosure	3	2.54	3.46
Not share reviewers' comments with all co-authors	2.31	1.88	2.81

Social sciences researchers, μ, bootstrapped Bca 95% CI Lower Bound (LB), Upper Bound (UB)	μ	CI LB	CI UB
Insufficiently supervise or mentor junior coworkers	6.95	6.13	7.78
Not publish a valid 'negative' study	6.53	5.71	7.29
Let own convictions influence the conclusions substantially	5.86	5.24	6.48
Choose a clearly inadequate research design or using evidently unsuitable measurement instruments	5.77	5.06	6.51
Give insufficient attention to the equipment, skills or expertise which are essential to perform the study	5.71	5.06	6.42
Report an unexpected finding as having been hypothesized from the start	5.6	4.94	6.25
Selectively cite to enhance own findings or convictions	5.45	4.96	5.95
Keep inadequate notes of the research process	5.4	4.8	6.04
Ignore basic principles of quality assurance	5.35	4.78	5.99
Conceal results that contradict earlier findings or convictions	5.33	4.88	5.85
Not report clearly relevant details of study methods	5.33	4.69	5.96
Unfairly review papers, grant applications or colleagues applying for promotion	5.28	4.64	5.91
Not report replication problems	5.23	4.68	5.82
Insufficiently report study flaws and limitations	5.23	4.56	5.9
Make no clear distinction between personal views and professional comments	5.19	4.53	5.95
Selectively cite to please editors, reviewers or colleagues	5.05	4.32	5.92
Not report all study protocol-stipulated results	5.04	4.4	5.68
Perform data-analyses not stated in the study protocol without disclosure	5.02	4.51	5.59
Selectively delete data, modify data or add fabricated data after performing initial data-analyses	5.01	4.6	5.35
Report on data-driven hypotheses without disclosure	4.85	4.25	5.44
Turn a blind eye to putative breaches of research integrity by others	4.82	4.27	5.42
Fabricate data	4.8	4.54	5.1
Modify the results or conclusions of a study due to pressure of a sponsor	4.78	4.51	5.09
Deliberately communicate findings inaccurately in the media or during presentations	4.62	4.16	5.14
Propose study questions which are clearly irrelevant	4.53	3.84	5.35
Delete data before performing data analysis without disclosure	4.47	4.09	4.87
Collect more data after noticing that the results are almost statistically significant	4.42	3.87	5.03
Write no or a clearly inadequate research protocol	4.4	3.84	4.99
Inadequately handle or store data or materials	4.33	3.72	5.03
Importantly change the research design during the study without disclosure	4.3	3.87	4.74
Spread study results over more papers than needed	4.26	3.66	4.89
Demand or accept an authorship without significant contribution	4.23	3.65	4.82
Selectively cite or cite own work to improve citation metrics	4.22	3.75	4.73
Refuse to respond to an allegation of a breach of research integrity	4.16	3.87	4.48
Take no full responsibility for the integrity of the research project and its reports	4.1	3.6	4.73
Review one's own submitted manuscripts	4.1	3.77	4.47
Communicate results to the general public before a peer reviewed publication is available	4.1	3.63	4.62
Refuse to share data with bona fide colleagues	4.08	3.61	4.6
Stop data collection earlier than planned because the results are already statistically significant	4.01	3.61	4.49
Failure to disclose a relevant financial or intellectual conflict of interest	3.96	3.54	4.38
Use published ideas or phrases of others without referencing	3.93	3.49	4.43
Handle existing conflicts of interest inadequately	3.75	3.27	4.22
Report an incorrect downwardly rounded p-value	3.71	3.38	4.08
Ignore substantial risks of the expected findings for society or environment	3.7	3.26	4.22
Gross unfairness to collaborators	3.65	3.12	4.22
Ignore substantial safety risks of the study to participants, workers or environment	3.6	3.23	4
Present grossly misleading information in a grant application	3.56	3.11	4
Re-use of previously published data without disclosure	3.54	3.06	4.1
Not adhere to pertinent laws and regulations	3.53	3.25	3.83
Add an author who doesn't qualify for authorship	3.4	2.99	3.87
Use unpublished ideas or phrases of others without their permission	3.36	2.98	3.74
Not ask permission from contributors for the wording of the acknowledgement	3.34	3	3.73
Failure to disclose a sponsor of the study	3.31	2.98	3.36
Omit a contributor who deserves authorship	3.31	2.89	3.75
Duplicate publication without disclosure	3.28	2.97	3.6
Demand, accept or offer substantial gifts for doing a favour	3.24	2.96	3.5
Re-use parts of own publications without referencing	3.2	2.81	3.57
Submit or resubmit a paper or grant application without consent from all authors	3.04	2.67	3.43
Not acknowledge contributors who do not qualify for authorship	2.99	2.6	3.4
Not share reviewers' comments with all co-authors	2.45	2.15	2.79

Humanities researchers, μ, bootstrapped Bca 95% CI Lower Bound (LB), Upper Bound (UB)	μ	CI LB	CI UB
Insufficiently supervise or mentor junior coworkers	6.76	5.7	7.74
Let own convictions influence the conclusions substantially	6.69	5.46	8
Selectively cite to enhance own findings or convictions	6.17	5.17	7.23
Choose a clearly inadequate research design or using evidently unsuitable measurement instruments	6.11	5.11	7.14
Selectively cite to please editors, reviewers or colleagues	6.03	5.13	7.03
Unfairly review papers, grant applications or colleagues applying for promotion	6.03	4.77	7.41
Make no clear distinction between personal views and professional comments	5.68	4.57	6.82
Insufficiently report study flaws and limitations	5.42	4.64	6.14
Ignore basic principles of quality assurance	5.27	4.48	6.03
Deliberately communicate findings inaccurately in the media or during presentations	5.24	4.11	6.35
Demand or accept an authorship without significant contribution	5.22	4.22	6.25
Use published ideas or phrases of others without referencing	5.07	4.07	6.21
Fabricate data	4.91	4.35	5.59
Conceal results that contradict earlier findings or convictions	4.82	4.18	5.47
Handle existing conflicts of interest inadequately	4.71	3.98	5.53
Turn a blind eye to putative breaches of research integrity by others	4.67	3.59	5.69
Propose study questions which are clearly irrelevant	4.64	3.58	5.74
Keep inadequate notes of the research process	4.5	3.71	5.32
Selectively delete data, modify data or add fabricated data after performing initial data-analyses	4.46	3.91	5
Modify the results or conclusions of a study due to pressure of a sponsor	4.44	3.97	4.9
Importantly change the research design during the study without disclosure	4.41	3.81	5.04
Not adhere to pertinent laws and regulations	4.24	3.81	4.73
Re-use parts of own publications without referencing	4.24	3.55	5.06
Report an unexpected finding as having been hypothesized from the start	4.21	3.36	5.1
Not publish a valid 'negative' study	4.2	3.5	5
Use unpublished ideas or phrases of others without their permission	4.19	3.16	5.31
Selectively cite or cite own work to improve citation metrics	4.13	3.12	5.32
Present grossly misleading information in a grant application	4.12	3.71	4.65
Write no or a clearly inadequate research protocol	4.1	3.41	4.86
Spread study results over more papers than needed	4.09	3.25	5
Gross unfairness to collaborators	4.03	3.12	5.03
Not report replication problems	4	3.1	5.12
Not report all study protocol-stipulated results	3.98	3.46	4.51
Refuse to respond to an allegation of a breach of research integrity	3.91	3.47	4.38
Review one's own submitted manuscripts	3.9	3.38	4.37
Give insufficient attention to the equipment, skills or expertise which are essential to perform the Stu	3.9	3.03	4.9
Stop data collection earlier than planned because the results are already statistically significant	3.88	3.31	4.55
Report on data-driven hypotheses without disclosure	3.85	3.44	4.27
Collect more data after noticing that the results are almost statistically significant	3.77	3.21	4.46
Communicate results to the general public before a peer reviewed publication is available	3.76	3.27	4.27
Perform data-analyses not stated in the study protocol without disclosure	3.75	3.08	4.56
Demand, accept or offer substantial gifts for doing a favour	3.75	3.33	4.17
Omit a contributor who deserves authorship	3.74	3.13	4.39
Take no full responsibility for the integrity of the research project and its reports	3.7	3.19	4.21
Inadequately handle or store data or materials	3.68	3.01	4.34
Add an author who doesn't qualify for authorship	3.65	2.81	4.61
Refuse to share data with bona fide colleagues	3.62	2.91	4.38
Re-use of previously published data without disclosure	3.59	3.06	4.11
Ignore substantial risks of the expected findings for society or environment	3.55	2.67	4.61
Delete data before performing data analysis without disclosure	3.5	2.98	4.02
Not report clearly relevant details of study methods	3.41	2.88	3.97
Duplicate publication without disclosure	3.22	2.69	3.78
Failure to disclose a relevant financial or intellectual conflict of interest	3.19	2.74	3.71
Not ask permission from contributors for the wording of the acknowledgement	3.1	2.83	3.35
Failure to disclose a sponsor of the study	3.08	2.68	3.45
Not acknowledge contributors who do not qualify for authorship	3.05	2.42	3.78
Report an incorrect downwardly rounded p-value	3.03	2.55	3.55
Submit or resubmit a paper or grant application without consent from all authors	2.92	2.47	3.4
Ignore substantial safety risks of the study to participants, workers or environment	2.68	2.13	3.36
Not share reviewers' comments with all co-authors	2.58	2.21	3.32
