# Supplementary material for: Researchers’ perceptions of research misbehaviours: a mixed methods study among academic researchers in Amsterdam
Source: Res Integr Peer Rev. 2019 Dec 2;4:25. doi: 10.1186/s41073-019-0081-7 (PMC6886174; doi:10.1186/s41073-019-0081-7)
Supplement: Supplementary file 12 — Additional file 12. Qualitative rankings of research misbehaviours according to the focus group participants. * Italics denote research misbehaviours that came from the quantitative survey. Focus groups were conducted over 3 academic ranks (see rows, PhD students, postdocs and assistant professors and associate and full professors) and 4 academic ranks (see columns, biomedical sciences, natural sciences, social sciences and humanities). The higher the misbehaviour in the rows; the more detrimental focus group participants indicated this research misbehaviour to be. [file 41073_2019_81_MOESM12_ESM.pdf]

Additional file 12. Qualitative rankings of detrimental research misbehaviours per focus group.

**Detrimental research misbehaviours stratified per academic rank and disciplinary field**			
**Biomedical sciences researchers**	**Natural sciences researchers**	**Social sciences researchers**	**Humanities researchers**
**PhD students**	**PhD students**	**PhD students**	**PhD students**
Bad administration/no raw data	No mechanism for reporting errors in already published papers	*Let own convictions influence conclusions substantially*	Discrimination
Inadequate power analysis	Hiding important information from the data with wrong statistical test (intentionally or unintentionally)	An emphasis on what is known instead of stimulating the creative process of thinking outside the box	Manipulation by stakeholder
Choose a clearly inadequate research design or using evidently unsuitable measurement instruments	*Insufficiently supervising or mentoring junior co-workers*	Publication pressure preventing proper skill- and knowledge development by putting timeframes on writing dissertation & writing & reviewing papers	Intentional destruction of artefacts
Selectively choosing primary outcome study	Changing the parameters in the method, not to allow the other scientists reproduce the work quickly	Time management, publication and teaching obligation spiral in the tenure track	Guest authorship

Keep inadequate notes of the research process	Withholding material for reproduction of published work	Peer reviewing seems overly critical, we are trained to see what is "bad" instead of seeing what is "good". Especially in small fields with same reviewers for different journals, a reviewer has extensive power	*Unfairly review papers, grants, colleagues applying for promotion*
**Postdocs and assistant professors**	**Postdocs and assistant professors**	**Postdocs and assistant professors**	**Postdocs and assistant professors**
Let own convictions influence conclusions	Strengthening your own position through power abuse	Bullying juniors	Stealing someone else's PhD (allowing them to finish and defend)
Considering an opinion as more valuable than objective indicators of good research	A senior research that gets too many PhD candidates and postdocs; group relations may sour and leads to the PhD candidates not getting adequate supervision	Pressuring junior postdocs and PhDs in "finding" significant effects	Taking PhD students work for publication without acknowledgement/ Using students' work for publication with little or no acknowledgement
Investigating the null hypothesis less thoroughly than the alternative hypothesis	Inadequate supervision (linked to following items)	Pressure to work on grants while you are still working on your former project (PhD project)	Submitting publishing the same material (text) in different journals chapters

Lack of doubt	Writing a manuscript chronologically	Adjust covariates to get good results/ Adjust correction for multiple testing	Sending PhD students here for supervision (from abroad, since actual supervisor is not an expert)
Getting too little time for research from mentor	Failing to attribute own success to team effort	Supervisors exploiting PhDs/postdocs via their power positions (co-authorships/ harassments)	*Insufficiently supervise or mentor junior co-workers*
**Associate and full professors**	**Associate and full professors**	**Associate and full professors**	**Associate and full professors**
Wrong role models	Falsifying results	Not carrying out replication study/ not reporting of non-replication	Plagiarism
Insufficiently supervise or mentor junior co-workers	Plagiarism	*Choose a clearly inadequate research design or evidently unsuitable measurement instruments*	Reviewer rejecting paper and then stealing ideas
Not being transparent in reporting/ keep inadequate notes of research process	Declining or withholding publication by reviewing competitor	Report an unexpected finding as being hypothesised from the start	Data manipulation (adjustment analyses)
Not publishing results	Abuse of referee or editor power/ Referees that steal	Selective data reporting	Making up data

	ideas from proposals/manuscripts they need to evaluate		
Leaving out data/Selectively including/excluding data	Espionage/ stealing (conscious or unconscious)	Publication bias	Ideologizing science / Economising science / Politicalising science

* *Italics* denote research misbehaviours that came from the quantitative survey. Focus groups were conducted over 3 academic ranks (see rows, PhD students, postdocs and assistant professors and associate and full professors) and 4 academic ranks (see columns, biomedical sciences, natural sciences, social sciences and humanities). The higher the misbehaviour in the rows; the more detrimental focus group participants indicated this research misbehaviour to be.
