# Supplementary material for: Researchers’ perceptions of research misbehaviours: a mixed methods study among academic researchers in Amsterdam
Source: Res Integr Peer Rev. 2019 Dec 2;4:25. doi: 10.1186/s41073-019-0081-7 (PMC6886174; doi:10.1186/s41073-019-0081-7)
Supplement: Supplementary file 13 — Additional file 13. List of 60 misbehaviours with alternative formulations and newly added items from the focus group interviews printed in italics. Newly added and preferred formulations of existing items are printed italic-bold and the former formulation is crossed out. [file 41073_2019_81_MOESM13_ESM.pdf]

Additional file 13. List of 60 misbehaviours with alternative formulations and newly added items from the focus group interviews printed in *italics*. Newly added and preferred formulations of existing items are printed ***italic-bold*** and the former formulation is ~~crossed out.~~

Study design

1. Propose study questions which are clearly irrelevant
2. Choose a clearly inadequate research design or using evidently unsuitable measurement instruments
3. Present grossly misleading information in a grant application

   Overpromising future research results in a grant application (knowing it's impossible to obtain these results)
4. Write no or a clearly inadequate research protocol
5. Ignore substantial safety risks of the study to participants, workers or environment
6. Ignore substantial risks of the expected findings for society or environment
7. Importantly change the research design during the study without disclosure
8. Give insufficient attention to the equipment, skills or expertise which are essential to perform the study

   Not keeping up with scientific developments in your field

Data collection

9. Collect more data after noticing that the results are almost statistically significant
10. Fabricate data

    Make up data
11. Stop data collection earlier than planned because the results are already statistically significant
12. Not adhere to pertinent laws and regulations

13. Inadequately handle or store data or materials

    Un-anonymised data storage without consent

14. Keep inadequate notes of the research process

15. Ignore basic principles of quality assurance

16. *Publish datasets unorganised in an Open Access database that are not usable for interested researchers*

17. ***Violating medical ethical guidelines or privacy guidelines***

18. *Inadequate or no power analysis*

    To not conduct a power calculation or sample size calculation, although required

    Insufficient attention to statistical power in the research design

    Change intended sample size after data collection without disclosure

---

Reporting

---

19. Report on data-driven hypotheses without disclosure

    Hedging of chances by including more variables (dependent or independent) than the primary study entails in their research protocol

    Changing the research question after results are known

20. Delete data before performing data analysis without disclosure

21. Selectively delete data, modify data or add fabricated data after performing initial data-analyses

    Sample exclusion or extension to obtain the desired outcome

    Searching for a statistical test or statistical package that gives the most significant result

    Hiding important information from the data with wrong statistical test

    Deleting or removing some part of the data set to get a nice curve or a nice result

    Intentional destruction of archaeological or otherwise historical artefacts

Adjust covariates to get positive results

Selectively including/excluding data without disclosure

Adjust the study's experimental power until the desired effect can be reported

Intentionally misinterpreting results from a statistical analysis

22. ~~Perform data-analyses not stated in the study protocol without disclosure~~

    Perform post-hoc analyses without disclosure

23. Report an incorrect downwardly rounded p-value
24. Not report all study protocol-stipulated results

    Failing to disclose all measures collected in a study

    Selectively choosing primary outcome study

    Present secondary outcomes as primary if initial primary outcome gives no or little result

25. Not publish a valid 'negative' study

    Only considering to publish a study when the results are negative

    Not publishing results

26. Report an unexpected finding as having been hypothesized from the start
27. Conceal results that contradict earlier findings or convictions
28. Not report clearly relevant details of study methods

    Changing the parameters in the method so that other researchers cannot reproduce your results

    Not sharing all details relevant for reproducing experimental results

    Not being transparent in reporting

29. ~~Not report replication problem~~

    Not reporting of failure to replicate

30. Selectively cite to enhance own findings or convictions

Intentionally use a selective bibliography

31. Selectively cite to please editors, reviewers or colleagues

    Referees that impose citations to their own work

32. Selectively cite or cite own work to improve citation metrics
33. Let own convictions influence the conclusions substantially

    Treating an opinion as more valuable than evidence from adequate research.

    Lack of doubt or being too convinced about own research to soundly judge its merit

    Flexible interpretation of the literature so it fits your hypothesis

34. Insufficiently report study flaws and limitations
35. Spread study results over more papers than needed

    Salami slicing: To split one research question/data set into multiple articles to publish more with the same work

    Cutting up data and spreading it over as many publications as possible

36. Duplicate publication without disclosure

    Submitting/publishing the same material (text) in different journals chapters

37. Re-use parts of own publications without referencing

    Self-plagiarism

38. Re-use of previously published data without disclosure

    "Double publishing" of similar research more than once

39. Modify the results or conclusions of a study due to pressure of a sponsor ***or other stakeholder***
40. Failure to disclose a sponsor of the study
41. Failure to disclose a relevant financial or intellectual conflict of interest
42. Handle existing conflicts of interest inadequately

43. Communicate results to the general public before a peer reviewed publication is available

44. Deliberately communicate findings inaccurately in the media or during presentations

    Intentionally misrepresenting the scientific literature to the public

45. Make no clear distinction between personal views and professional comments

46. *Investigating the null hypothesis less thoroughly than the alternative hypothesis*

47. Exaggerate experimental results

    Interpreting results inadequately and present them as spectacular effects with little critical evaluation

    Oversell research as more than it is

48. No critical appraisal of negative aspects of novel methodology

49. Writing a paper too fast without proper and rigorous analysis

    Claiming that something is new or offers a new perspective without proper reading

50. ***Change a manuscript due to pressure from editors or reviewers in a grossly incorrect way in order to get it published***

51. *Overutilization of databases*

---

52. *Exclusion of outliers without disclosure*

Deleting or removing some part of the data set to get a nice curve or a nice result

---

Collaboration

---

53. Take no full responsibility for the integrity of the research project and its reports

54. Refuse to share data with bona fide colleagues

    Withholding data for reproduction of published work

    Treating data as if you "owned" it and other researchers have no rights to use that data

55. Turn a blind eye to putative breaches of research integrity by others

56. Refuse to respond to an allegation of a breach of research integrity

57. Use unpublished ideas or phrases of others without their permission

    Referees that steal ideas from proposals or manuscripts they need to evaluate

    As a reviewer rejecting a paper and then stealing the ideas from that paper

    Taking PhD students' or students' work for publication without permission/acknowledgement

58. Use published ideas or phrases of others without referencing

    Steal data or copy findings without consent

    Plagiarism

59. Unfairly review papers, grant applications or colleagues applying for promotion

    Nepotism in grant funding, publication review, promotion, etc.

    To accept papers for conferences or articles for proceedings based on names or someone's status in the field

    Feeling you are obliged to accept a bad paper because it is someone's PhD student

    Declining or withholding publication from a competitor

    Abuse of referee power

60. Review one's own submitted manuscripts

61. Demand, accept or offer substantial gifts for doing a favour

62. Insufficiently ***or inadequately*** supervise or mentor junior coworkers

    Knowingly supervising a PhD student whilst there is not sufficient knowledge on their topic in the research group and failing to direct them elsewhere

    Pressuring junior postdocs and PhDs in "finding" significant effects

    Pressure your PhD student to work on other research projects while PhD student is still working on former (PhD) project

Not provide a safe learning environment where they are allowed to learn and make mistakes for more junior researchers

Supervisors writing (part of) chapters for PhD students to guarantee graduation

Intentionally wrong role modelling for junior colleagues to create a culture of fear

63. Gross unfairness to collaborators

    Failing to attribute own success to team effort

    Not doing your fair share in collaborations

64. Add an author who doesn't qualify for authorship

    Guest authorship

    First authorship or co-authorship without doing significant work

65. Demand or accept an authorship without significant contribution
66. Omit a contributor who deserves authorship

    Ghost writing a paper for someone else

67. Not acknowledge contributors who do not qualify for authorship
68. Not ask permission from contributors for the wording of the acknowledgement
69. Not share reviewers' comments with all co-authors
70. Submit or resubmit a paper or grant application without consent from all authors
71. ***Misuse of research grants for unintended purposes***
